# Identification of three new isolates of *Tomato spotted wilt virus* from different hosts in China: molecular diversity, phylogenetic and recombination analyses

**DOI:** 10.1186/s12985-015-0457-3

**Published:** 2016-01-14

**Authors:** Zhenjia Zhang, Deya Wang, Chengming Yu, Zenghui Wang, Jiahong Dong, Kerong Shi, Xuefeng Yuan

**Affiliations:** College of Plant Protection, Shandong Agricultural University, No 61, Daizong Street, Tai’an, 271018 Shandong Province P. R. China; Resources Institute, Yunnan Academy of Agricultural Sciences, Yunnan Provincial Key Lab of Agricultural Biotechnology, Key Lab of Southwestern Crop Gene Resources and Germplasm Innovation, Ministry of Agriculture, Kunming, 650223 China; College of Animal Science and Technology, Shandong Agricultural University, Tai’an, 271018 P. R. China

**Keywords:** *Tomato spotted wilt virus*, Phylogenetic analysis, Reassortment, Recombination

## Abstract

**Background:**

Destructive diseases caused by *Tomato spotted wilt virus* (TSWV) have been reported associated with many important plants worldwide. Recently, TSWV was reported to infect different hosts in China. It is of value to clone TSWV isolates from different hosts and examine diversity and evolution among different TSWV isolates in China as well as worldwide.

**Methods:**

RT-PCR was used to clone the full-length genome (L, M and S segments) of three new isolates of TSWV that infected different hosts (tobacco, red pepper and green pepper) in China. Identity of nucleotide and amino acid sequences among TSWV isolates were analyzed by DNAMAN. MEGA 5.0 was used to construct phylogenetic trees. RDP4 was used to detect recombination events during evolution of these isolates.

**Results:**

Whole-genome sequences of three new TSWV isolates in China were determined. Together with other available isolates, 29 RNA L, 62 RNA M and 66 RNA S of TSWV isolates were analyzed for molecular diversity, phylogenetic and recombination events. This analysis revealed that the entire TSWV genome, especially the M and S RNAs, had major variations in genomic size that mainly involve the A-U rich intergenic region (IGR). Phylogenetic analyses on TSWV isolates worldwide revealed evidence for frequent reassortments in the evolution of tripartite negative-sense RNA genome. Significant numbers of recombination events with apparent 5′ regional preference were detected among TSWV isolates worldwide. Moreover, TSWV isolates with similar recombination events usually had closer relationships in phylogenetic trees.

**Conclusions:**

All five Chinese TSWV isolates including three TSWV isolates of this study and previously reported two isolates can be divided into two groups with different origins based on molecular diversity and phylogenetic analysis. During their evolution, both reassortment and recombination played roles. These results suggest that recombination could be an important mechanism in the evolution of multipartite RNA viruses, even negative-sense RNA viruses.

**Electronic supplementary material:**

The online version of this article (doi:10.1186/s12985-015-0457-3) contains supplementary material, which is available to authorized users.

## Background

Tomato spotted wilt disease caused by *Tomato spotted wilt virus* (TSWV) was first described in 1919 in Australia (Brittlebank, 1919) [1] and has gradually spread to Europe, Africa, North America, and South America [2–11]. Recently, TSWV was detected in middle-eastern and far-eastern countries in Asia including Iran, Japan, South Korea and China [12–17]. TSWV is mainly transmitted in a persistent manner by several species of thrips, especially the western flower thrip *Frankliniella occidentalis,* which may contribute to the worldwide spread of TSWV as well as to tomato spotted wilt disease [14, 18, 19]. TSWV infectes many important economic plants including tomato and potato, and causes serious damage including ringspot, black streak and tip dieback [20–22].

**Fig. 1:**
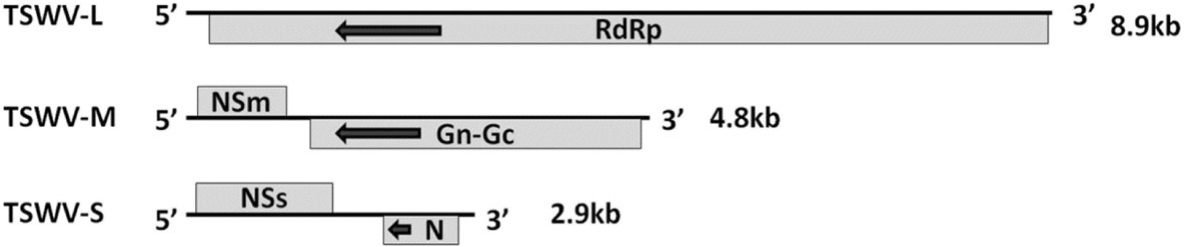
Genomic organization of *Tomato spotted wilt virus.* RdRp: RNA-dependent RNA polymerase; NSm: nonstructural protein encoded by RNA M; Gn-Gc: Gn-Gc glycoprotein; NSs: nonstructural protein encoded by RNA S; N: nucleocapsid protein. Left pointing arrows denote that the ORF is on the viral complementary strand

TSWV is the type member of the genus *Tospovirus* in the family *Bunyaviridae* [23]. TSWV has three single-stranded RNA segments denoted as L (8.9 kb), M (4.8 kb) and S (2.9 kb), which together encode five proteins (Fig. 1). RNA L is a negative-sense RNA, whose complementary strand encodes the RNA-dependent RNA polymerase (RdRp), which is required for viral replication [24]. RNA M and S are ambisense with two ORFs, one expressed from the viral sense and the other the viral complementary strand. M plus-sense [M(+)RNA] encodes a nonstructural protein (NSm) responsible for cell-to-cell movement and its complementary minus-strand [M(−)RNA] encodes a Gn-Gc glycoprotein [25]. S(+)RNA encodes a second nonstructural protein (NSs) involved in the suppression of gene silencing and S(−)RNA encodes the nucleocapsid (N) protein [26].

To date, whole-genome sequences have been determined for 19 TSWV isolates including 14 from South Korea, two from China (YN and CG-1), 1 from Brazil (BR-01) and 2 from Italy (p202/3WT and p105) (Additional file 1: Table S1) [16, 17, 24]. There are currently 26 full-length L, 59 full-length M, and 63 full-length S sequences deposited in the database (Additional file 1: Table S1). Previous studies suggested that both reassortment and recombination have contributed to the molecular diversity and evolution of TSWV based on partial sequences or regional whole-genome isolates [17, 27, 28]. However, only a few recombination events associated with TSWV have been described [17].

To identify the origin and evolution of TSWV in China, whole-genome sequences of three new TSWV isolates from tobacco, red pepper and green pepper in China were determined in this study. Molecular diversity and phylogenetic analysis revealed that all five Chinese TSWV isolates can be divided into two groups. Reassortment and huge numbers of recombination events were found based on phylogenetic and recombination analysis among all TSWV isolates worldwide.

## Results

### Sequencing of three new full-length TSWV isolates and examination of molecular diversity of TSWV in China

Three new TSWV isolates (YNta, YNrp and YNgp) originating in three different infected plants (tobacco, red pepper and green pepper) were obtained in Yunnan province (China) in 2013. The genome information of these isolates (YNta, YNrp and YNgp) is listed in Additional file 1: Table S1, which also contains information on previously reported TSWV isolates. RNA L of YNta, YNrp and YNgp were identical lengths (8913 nts), while the genomic sizes of RNAs M and S varied slightly due to size variation in the intergenic region (IGR) (Additional file 1: Table S1).

When two other previously reported Chinese TSWV isolates (YN and CG-1) were included in the analysis, new characteristic of TSWV were observed. In addition to size variations in IGR of the RNA M and S, there were size variations in open reading frames (ORFs) and 5′ and 3′ untranslated regions (UTRs). For RNA L, the ORF in isolate CL4 (YN) is 8637 nt, compared with 8640 nt for other Chinese isolates. The 5′UTR and 3′UTR of isolate CL5 (CG-1) are 34 nt and 243 nt, respectively, compared with 33 nt and 240 nt in other Chinese isolates. These size variations contributed to the size differences among different isolates (CL4, 8910 nt; CL5, 8917 nt; CL1, CL2, CL3, 8913 nt). For RNA S, the 5′UTR of CS5 is 86 nt compared with 88 nt for other Chinese isolates. In addition, the size of the IGR in CS5 (503 nt) is shorter than the IGR in other Chinese isolates (548 to 551 nt) (Additional file 1: Table S1). Based solely on size similarities, TSWV isolates YNta, YNrp and YNgp may be more related than YN and CG-1.

In addition to genome size that reflects differences in ORFs and UTRs, nucleotide and amino acid sequences for the five Chinese TSWV isolates were also compared (Table 1). RNA L sequences were highly conserved, with nucleotide identity ranging from 99.17 to 99.52 %, whereas RNA S sequences were more variable (95.59 to 99.76 %). For amino acid sequences, the Gn-Gc protein was the most conserved (98.77 to 99.65 %), whereas NSs was more variable (97.05 to 99.79 %; Table 1). Among the five Chinese isolates, TSWV CG-1 was the most divergent (Table 1).

**Table 1:** Nucleotide and amino acid sequence identity for TSWV isolates in China

RNA L	CL1	CL2	CL3	CL4	CL5	
CL1		99.52	99.44	99.35	99.17	
CL2	99.17		99.46	99.35	99.20	
CL3	99.13	99.20		99.37	99.29	
CL4	97.43	97.53	97.46		99.18	
CL5	97.46	97.60	97.60	97.64		
RNA M	CM1	CM2	CM3	CM4	CM5	
CM1		99.29	99.41	99.62	97.97	
CM2	99.34(98.77)		99.29	99.31	97.74	
CM3	99.34(99.21)	99.34(98.85)		99.43	97.84	
CM4	99.34(99.65)	99.34(98.77)	99.34(99.21)		97.99	
CM5	98.01(99.56)	98.01(98.85)	98.01(99.30)	98.01(99.56)		
RNA S	CS1	CS2	CS3	CS4	CS5	CS6
CS1		99.43	99.56	99.43	95.63	99.56
CS2	98.72(98.84)		99.39	99.26	95.73	99.39
CS3	98.93(99.22)	98.93(99.61)		99.56	95.80	99.76
CS4	98.93(98.06)	98.93(98.45)	99.14(98.84)		95.59	99.60
CS5	97.00(98.45)	97.00(98.84)	97.64(99.22)	97.22(98.84)		95.86
CS6	99.14(98.84)	99.14(99.22)	99.79(99.61)	99.36(99.22)	97.86(99.61)	

CL1, CM1 and CS1 are the RNAs of TSWV isolate YNta; CL2, CM2 and CS2 are RNAs of TSWV isolate YNrp; CL3, CM3 and CS3 are RNAs of TSWV isolate YNgp; CL4, CM4 and CS4 are RNAs of TSWV isolate YN; CL5, CM5 and CS5 are RNAs of TSWV isolate CG-1; CS6 is another RNA of TSWV isolate KM-T

Percent identities were calculated using DNAMAN. Values above the diagonal shaded frames indicate the percentage of nucleotide sequence identity for L, M and S RNA, respectively. Values below the diagonal shaded frames indicate the percentage of amino acid sequence identity for RdRp, NSm (Gn-Gc) and NSs (N) encoded by L, M and S, respectively

### Molecular diversity and phylogenetic relationships among TSWV isolates worldwide

Whereas the TSWV Chinese isolates had slight differences in genome lengths when compared with each other, greater size differences were observed when all TSWV isolates were analyzed (Table 2). RNA L ranged from 8897 to 8917 nt, RNA M ranged from 4752 to 4830 nt, and RNA S ranged from 2916 to 3364 nt (Table 2). The size of ORFs of NSm, Gn-Gc and N were stable, whereas NSs and the RdRp ORFs varied by 204 nt and 15 nt, respectively. The IGRs in RNA M and S were highly variable in size, differing by 78 and 443 nt, respectively, whereas the 5′UTRs and 3′UTRs only had slight size variations (Table 2).

**Table 2:** Molecular diversity of RNA L, M and S for TSWV isolates worldwide

	RNA L	RNA M	RNA S
Genome size	8897–8917 (20)	4752–4830 (78)	2916–3364 (448)
ORF1	8628–8643 (15)	909 (0)	1200–1404 (204)
ORF2	None	3408 (0)	777 (0)
5′UTR	33–34 (1)	99–100 (1)	86–90 (4)
3′UTR	236–243 (7)	84–98 (14)	151–153 (2)
IGR	None	251–329 (78)	500–943 (443)

Values in brackets indicate the range of size variations (in nucleotides). ORF1 encodes the RdRp in RNA L, NSm is encoded by RNA M and NSs is encoded by RNA S. ORF2 encodes Gn-Gc in RNA M and N in RNA S. BD172070 (RNA L), HQ839730 (RNA S) and DQ431238 (RNA S) were not included in this analysis (Additional file 1: Table S1)

**Fig. 2:**
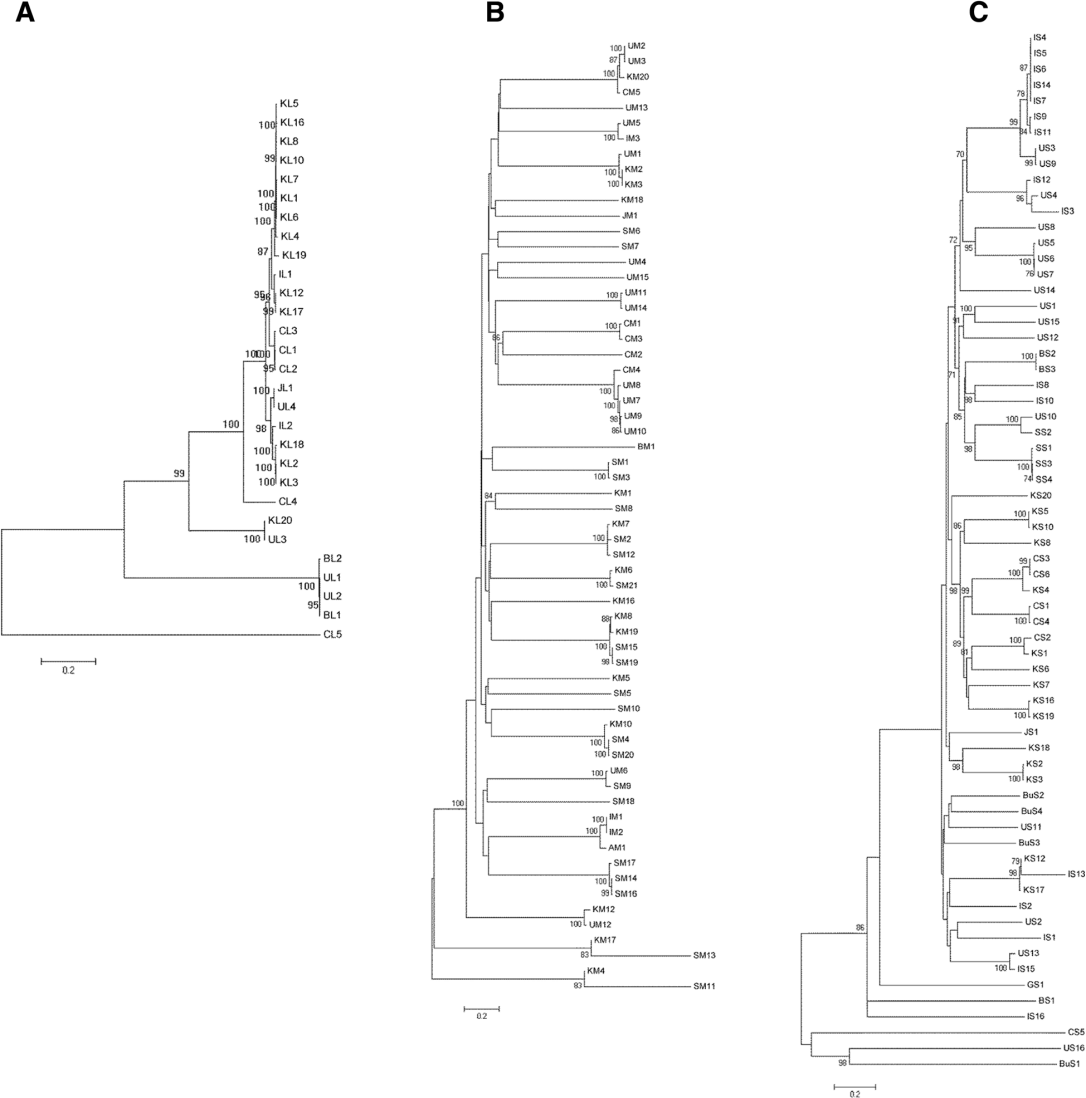
Phylogenetic trees of RNA L, M and S of TSWV isolates worldwide. **a** Phylogenetic tree of 29 full-length TSWV RNA L fragments, including CL1-CL5 from China, KL1-KL20 from South Korea, JL1 from Japan, UL1-UL4 from USA,BL1-BL1 from Brazil and IL1-IL2 from Italy. **b** Phylogenetic tree of 62 full-length TSWV RNA M fragments, including CM1-CM5 from China, KM1-KM20 from South Korea, JM1 from Japan, UM1-UM15 from USA, BM1 from Brazil, AM1 from Australia, SM1-SM21 from Spain and IM1-IM3 from Italy. **c** Phylogenetic tree of 66 full-length TSWV RNA S fragments, including CS1-CS6 from China, KS1-KS20 from South Korea, JS1 from Japan, US1-US16 from USA, BS1-BS3 from Brazil, SS1-SS4 from Spain, IS1-IS16 from Italy, GS1 from Germany and BuS1-BuS4 from Bulgaria. All phylogenetic trees were constructed using neighboring-joining (NJ) and Komura 2-parameter with bootstrap resampling (1000 replicates). The number at each branch of phylogenetic tree represents the bootstrap value (1000 replicates)

Phylogenetic trees based on the nucleotide sequences of RNA L, M and S were constructed. In the phylogenetic tree of RNA L, 29 isolates were divided into four clades (Fig. 2a). The first clade contained two sub-clades, one of which included a single Chinese isolate (CL4). The second sub-clade contained 21 isolates, inlcuding 14 from South Korea, three from China, one from Japan, two from Italy and 1 from the USA. The second clade contained only two isolates; KL20 from South Korea and UL3 from the USA (Fig. 2a). The third clade contained four isolates; two from Brail and two from the USA. The fourth clade contained a single Chinese isolate (CL5). The tree denoted that the evolutionary relationship and origin of the five Chinese isolates were divergent. CL1, CL2 and CL3 formed one branch, and were most closely related to most of the South Korean isolates. CL4 was in one clade and CL5 was the most divergent, since it formed a clade that was independent from the other 28 isolates (Fig. 2a).

In the phylogenetic tree of RNA M, 62 isolates were divided into three clades (Fig. 2b). The first clade included two sub-clades, one of which contained 56 isolates from South Korea, China, Japan, Italy, Spain, and the USA; the second sub-clade included two isolates from South Korea and the USA. Both the second and third clades contained isolates from South Korea and Spain (Fig. 2b). M RNAs of the five Chinese isolates were more related than their RNA L since they all belonged to the first sub-clade of the first clade. CM1, CM2 and CM3, which also form a branch, have the closest relationship with Chinese isolate CM4 and four isolates from the USA (UM7, UM8, UM9 and UM10). CM5 is most closely related to one isolate from South Korea (KM20) and two isolates from the USA (UM2 and UM3) (Fig. 2b). Therefore, Chinese isolate RNA M were most closely related to some TSWV isolates from USA.

In the phylogenetic tree of RNA S, 66 isolates were divided into two clades (Fig. 2c). The first clade included four sub-clades, the first one of which included 60 isolates from South Korea, China, Japan, Italy, Bulgaria, Brazil and USA; the second, third and fourth sub-clade contained one isolate from Germany (GS1), Brazil (BS1) and Italy (IS16) respectively. The second clade contained two sub-clades, one of which contained a single Chinese isolate (CS5), and the second contained one isolate from USA (US16) and one isolate from Bulgaria (BuS1) (Fig. 2c). The origin of RNA S of the 5 Chinese isolates differed from that of RNA L and M, with CS1, CS3 and CS4 comprising a branch with Chinese isolate CS6 and one isolate from South Korea (KS4). CS2 was most related to two isolates from South Korea (KS1 and KS6). CS5 formed a separate sub-clade in the second group (Fig. 2c). Therefore, with the exception of CS5, RNA S of Chinese isolates were most closely related to some TSWV isolates from South Korea.

Based on these phylogenetic trees, L, M and S of individual isolates could have different origins, implying the occurrence of frequent reassortment during the evolution of TSWV isolates. Among the Chinese isolates, RNA L (CL5) and S (CS5) of CG-1 were located in separate clades from the other four isolates, which correlated with the identity analysis of nucleotide sequence (Table 1). The three new isolates sequenced in this study (YNta, YNrp and YNgp) were more closely related to each other since their RNAs were contained in single branches in the phylogenetic trees of RNA L, M and S (Fig. 2a, b and c). These three new isolates also have a close relationship with isolate YN since they belonged to the same branch in phylogenetic trees of RNA M and S (Fig. 2b and c).

### Recombination analysis of TSWV isolates worldwide

**Fig. 3:**
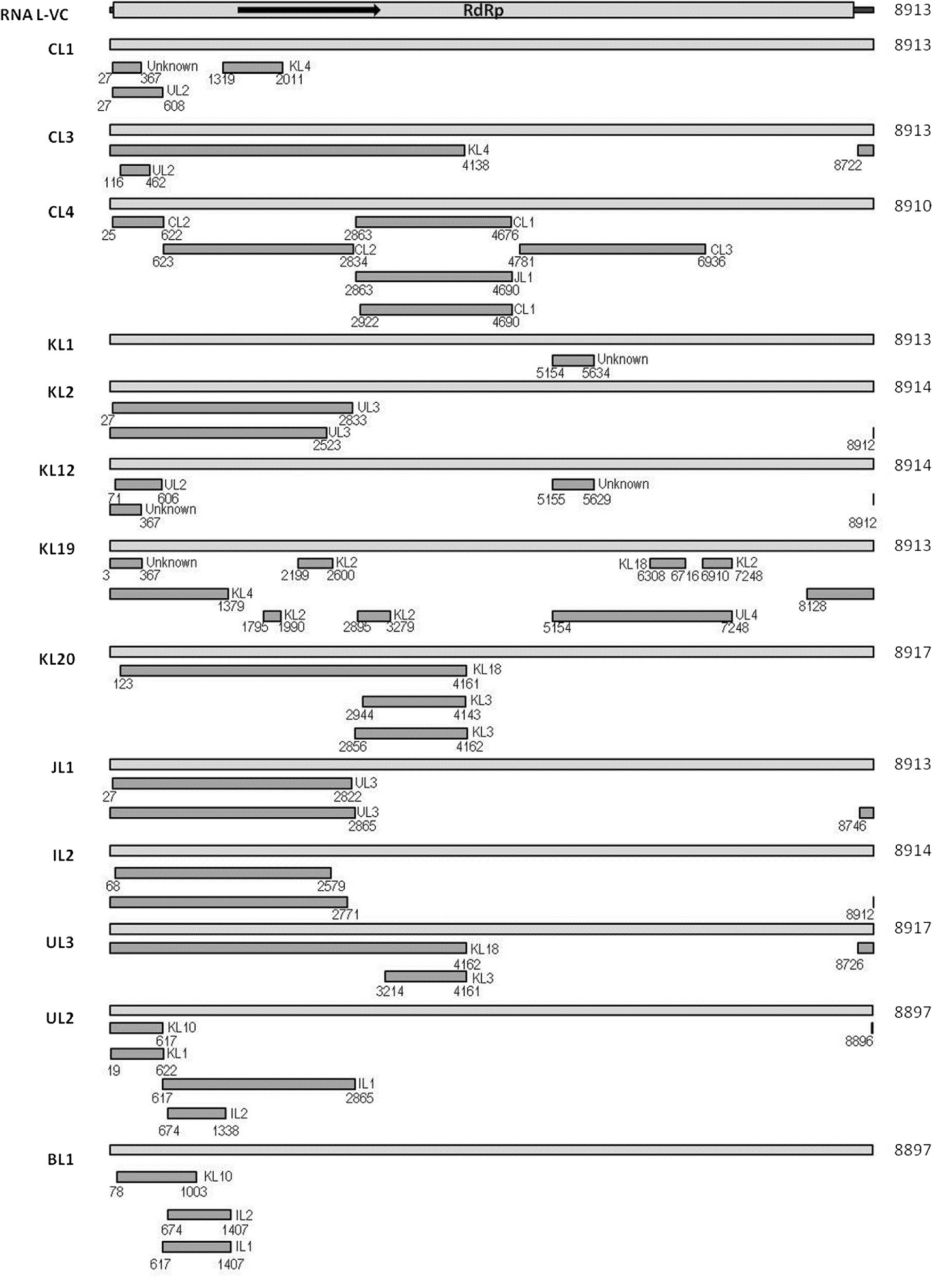
Analysis of putative recombination events in RNA L of TSWV isolates. Possible recombination events are indicated by black bars with minor parent and breakpoint positions of recombination sequences noted. Similar recombination events in different TSWV RNA L are shown once. Detailed information for each possible recombination event of TSWV RNA L is provided in Additional file 2: Table S2. “VC” indicates viral complementary strand

**Fig. 4:**
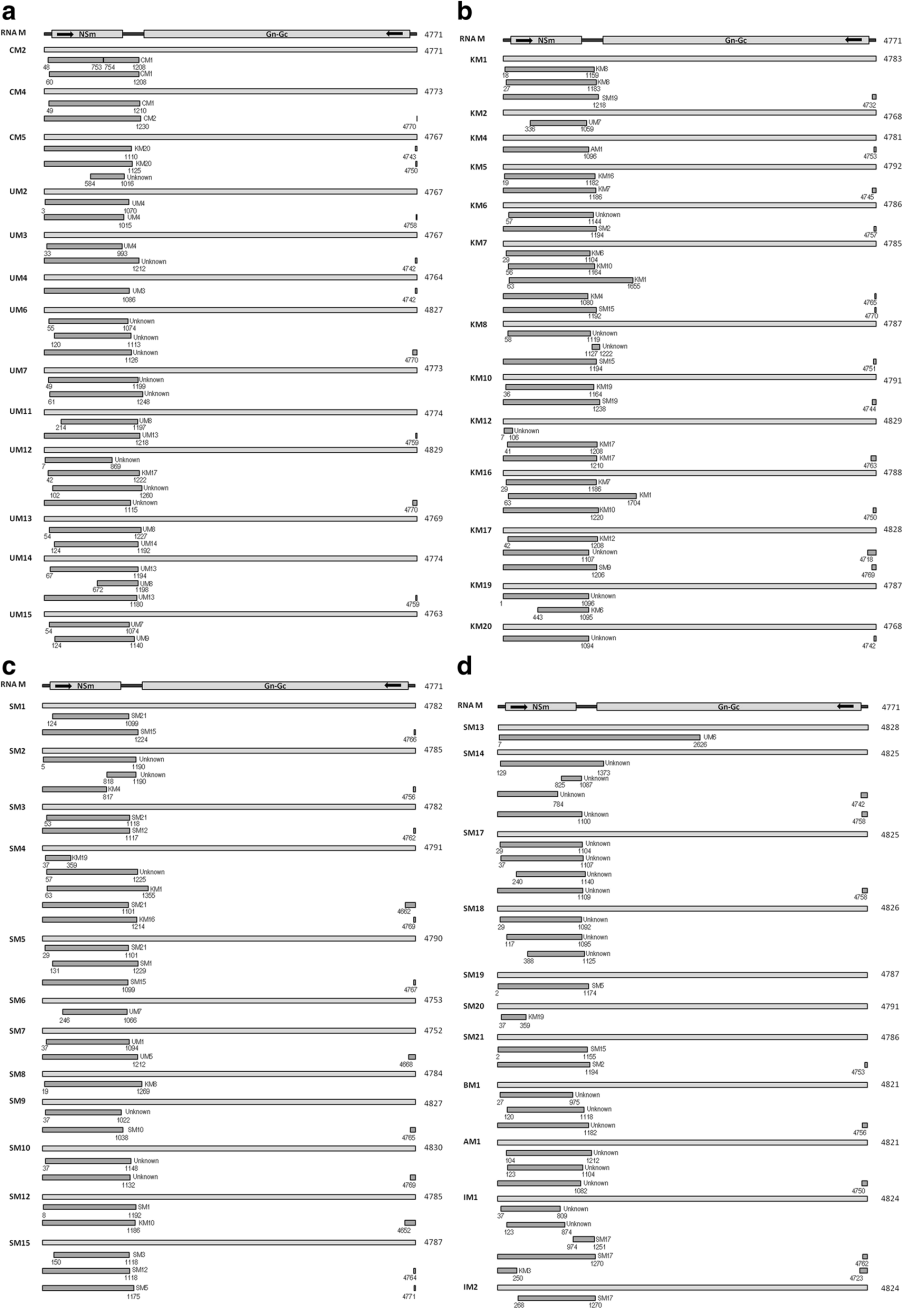
Analysis of possible recombination in RNA M of TSWV isolates. This figure includes 4 separate figures (4**a**, 4**b**, 4**c** and 4**d**) due to the large amount of recombination events. Detailed information for each recombination event is provided in Additional file 3: Table S3. See legend to Fig. 3 for additional information

**Fig. 5:**
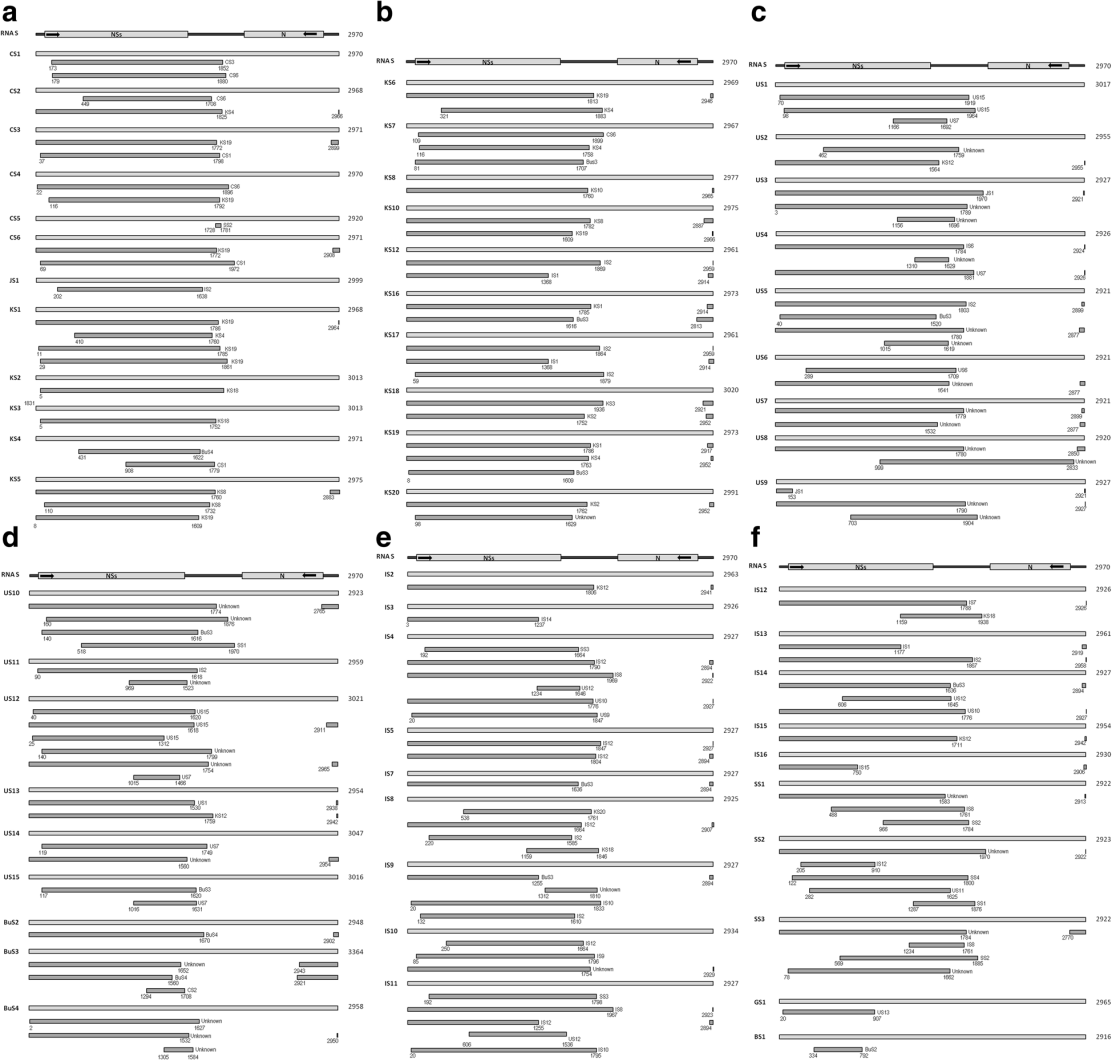
Analysis of possible recombination in RNA S of TSWV isolates. This figure includes 6 separate figures (5**a**, 5**b**, 5**c**, 5**d**, 5**e** and 5**f**) due to the large amount of recombination events. Detailed information for each recombination event is provided in Additional file 4: Table S4. See legend to Fig. 3 for additional information

As described above, the phylogenetic trees of RNA L, M and S of TSWVs suggest the occurrence of frequent reassortment during the evolution of TSWV. To further define possible mechanisms of TSWV evolution, RNA recombination was examined using the program RDP4. Surprisingly, sufficient numbers of recombination events were detected so that only events supported by at least three methods with P-values <1 × 10^−6^ were included in the analysis (Figs. 3, 4 and 5; Additional file 2: Tables S2, Additional file 3: Table S3 and Additional file 4: Table S4).

For 29 full-length RNA L fragments of TSWV, 88 recombination events were detected in 27 RNA L fragments (Additional file 2: Table S2). Only 2 TSWV RNA L (CL5 from China and BL2 from Brazil) did not have detectable recombination events. Recombination events in RNA L were located throughout the genome, although most were located in the 5′ half of the RdRp ORF (Fig. 3; Additional file 2: Table S2). Some short recombination events located in the 3′ terminal region are associated with recombination events in the 5′ half of the RdRp ORF, such as those located at 8722–4138 in CL3 (event 9), 8128–1379 in KL19 (event 15), 8746–2865 in JL1 (event 67), 8726–4162 in UL3 (event 77) etc. (Fig. 3 and Additional file 2: Table S2). In addition, separate recombination events that were located in the 3′ half of the RdRp ORF were detected in some RNA L from Chinese, South Korean and Italian isolates, such as recombination events located at 4781–6936 in CL4 (event 16), 5155–5629 in KL12, KL17 and IL1 (event 44, 50 and 85), 5154–5634 in KL1, KL4, KL6, KL7, KL8, KL10, KL16 and KL19 (event 19, 26, 29, 32, 35, 38, 41, 47 and 59), 5154–7248, 6308–6716 and 6910–7248 in KL19 (event 60–62) (Fig. 3 and Additional file 2: Table S2). Interestingly, recombination events having same breakpoints with same minor and major parents were detected in different RNA L, e.g., recombination events located at positions 5155–5629 (events 44/50/85) and ~8912-367 (events 42/49/86) found in KL12, KL17 and IL1 (Fig. 3 and Additional file 2: Table S2). Similar cases included events 69/73 and 70/72 in UL1 and UL2; events 68/71/80;events 1/4/7/17/24/27/30/33/36/39/45; events 2/5/18/25/28/31/34/37/40/46/54/84; events 20/22/51/79; events 64/75; events 43/48; events 19/26/32/35/38/41/47/59; events 6/9 and events 21/23/52 (detailed information are listed in Additional file 2: Table S2).

For 62 full-length TSWV RNA M fragments worldwide, 143 recombination events were totally detected from 56 RNA M fragments (Additional file 3: Table S3). Only in 6 RNA Ms (CM3, KM3 and KM18, JM1, UM1, SM11), no recombination event was detected. Preeminently within TSWV RNA M, recombination events are located at 5′ one-third of genome with only one exception, which occurred at position of 7–2626 in SM13 (event 99) (Fig. 4; Fig. 4d). In addition, there are three recombination events (including event 21, 33 and 99) bridging two ORF-coding regions (Fig. 4b and d). Similar to the recombination events of RNA L, many short recombination events of RNA M located at 3′ terminal region are simultaneously connected with those at 5′ one-third region of genome, such as recombination at 4743–1110 and 4750–1125 in CM5 (event 8–9), 4742–1212 for UM3 (event 45), 4770–1115 for UM12 (event 66) and so on (Fig. 4; Additional file 3: Table S3). However, different from the recombination events of RNA L, no separate recombination event was detected at the 3′ half of genomic RNA M. Recombination events with same breakpoints were also detected in different RNA M as the case of RNA L, for example, recombination located at position of 37–809 (events 133/138), 123–874 (events 134/139) and 974–1251(events 135/141) in IM1 and IM2 (Fig. 4 and Additional file 3: Table S3). Similar cases included events 82/122, 83/123 and 85/124 in SM4 and SM20; events 107/111, 108/112 and 110/114 in SM16 and SM17; events 5/53; events 51/56/60; events 52/54/57/58; events 55/70; events 1/4; events 101/109; events 42/45/47/142; events 137/143; events 105/119; events 6/59 (detailed information are listed in Additional file 3: Table S3).

For 66 full-length TSWV RNA S fragments, 174 recombination events were detected in 61 RNA S (Additional file 4: Table S4). Only 5 TSWV RNA S (US16, BS2 and BS3, IS1, BuS1) did not have detectable recombination events. Similar to the recombination events of RNA M, recombination events in TSWV RNA S were located within the 5′ 2000 nt of the RNA with one exception: position 999–2833 in US8 (event 70) (Fig. 5; Fig. 5c; Additional file 4: Table S5). Also, many short recombination events located in 3′ terminal regions were similarly associated with these 5′ region recombination events, such as 2899–1772 in CS3 (event 5), 2908–1772 in CS6 (event 10), 2883–1760 in KS5 (event 21), 2813–1616 in KS16 (event 35), 2943–1652 in BuS3 (event 96), etc. (Fig. 5; Additional file 4: Table S4). Furthermore, as found in RNAs L and M, recombination events with identical breakpoints were detected in different RNA S, e.g., recombination events located at position 78–1622, 1234–1761 and 2770–1784 in SS3 and SS4 (Fig. 5 and Additional file 4: Table S4). Similar cases included events 169/172, 167/171 and 166/170 in SS3 and SS4; events 109/115/121/126/154 and 104/110/117/123/149 in IS4, IS5, IS6, IS7 and IS14; events 58/63; events 80/87; events 62/66/68; events 107/113; events 112/119; events 33/37; events 161/106/111/118/124/132/141/150; events 108/120; events 116/122/155; events 84/91/93 (detailed information are listed in Additional file 4: Table S4).

**Fig. 6:**
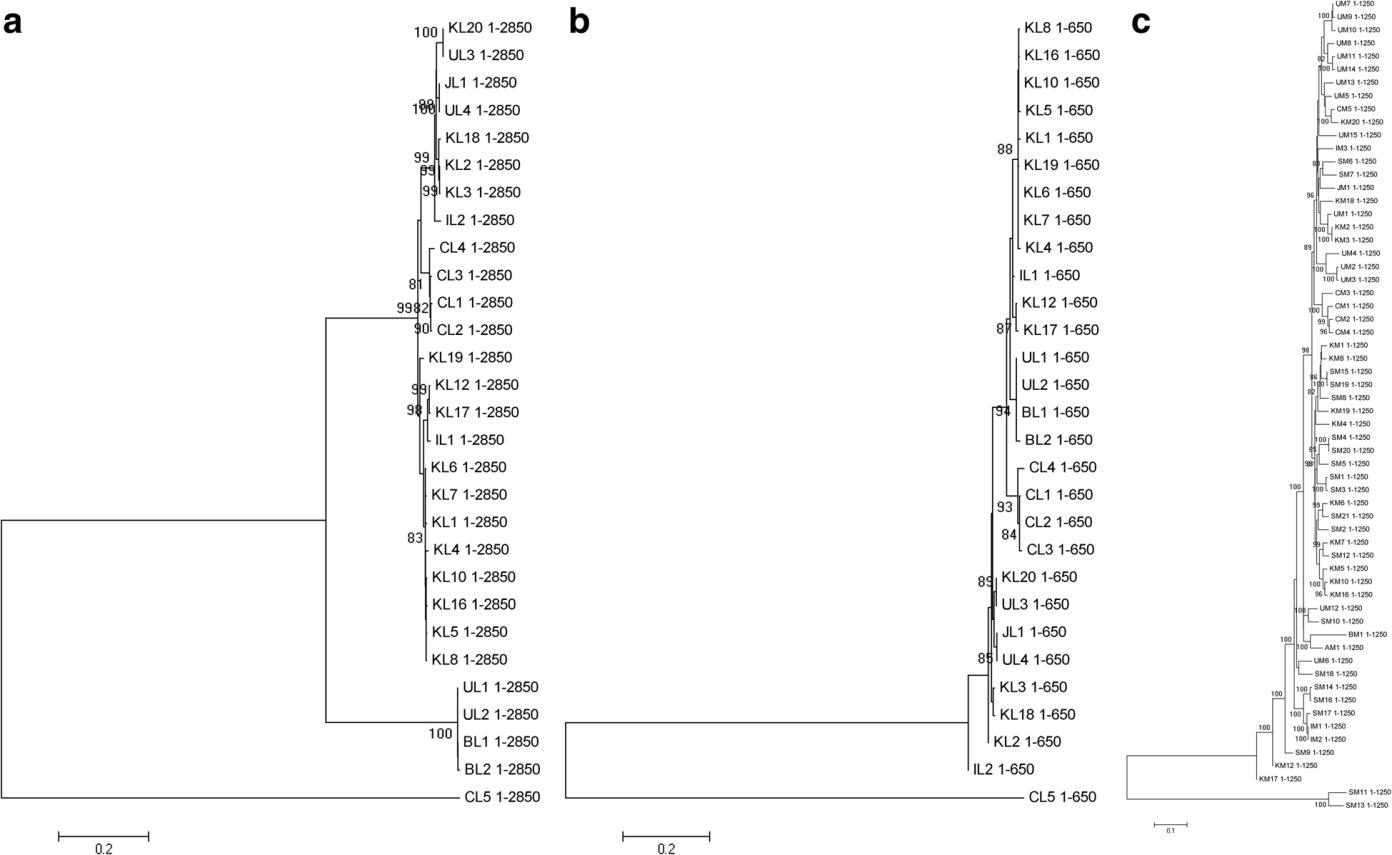
Phylogenetic trees based on fragments of RNA L and M of TSWV isolates. **a** Phylogenetic tree based on fragment 1–2850 of 29 RNA L. **b** Phylogenetic tree based on fragment 1–650 of 29 RNA L. **c** Phylogenetic tree based on fragment 1–1250 of 62 RNA M. See legend to Fig. 2 for additional information

To confirm the reliability of unusual frequent recombination events among TSWV in this study, phylogenetic trees based on recombination breakpoints were constructed to assess the relationship between the receptor and donor sequence. For RNA L, two phylogenetic trees based on fragment 1–2850 and 1–650 were constructed (Fig. 6a and b) since many recombination events have similar breaking points with these two regions (Fig. 3; Additional file 2: Table S2). For recombination among RNA L of TSWV, events 20/22/51/66/79 have similar breaking points (27–2833) in KL2, KL3, KL18, JL1 and UL4 (Additional file 2: Table S2), which were located in the same branch including their co-minor parent UL3, while their co-major parent KL16 belonged to the other further branch (Fig. 6a). It is suggested the intrinsic relationship between recombination events and phylogenetic trees, which confirmed the reliability of recombination events 20/22/51/66/79 (Fig. 6a; Additional file 2: Table S2). Events 2/5/18/25/28/31/37/40/46/54/84 have similar breaking points (27–606) with same minor and major parents (UL2/IL2) in CL1, CL2, KL1, KL4, KL5, KL6, KL8, KL10, KL16, KL19 and IL1 (Additional file 2: Table S2), which belonged to the same main branch including their co-minor parent UL2, while their co-major IL2 formed into a separate clade (Fig. 6b). It also implied the intrinsic relationship between recombination and phylogenetic analysis. For RNA M, one phylogenetic tree based on the fragment 1–1250 was constructed (Fig. 6c). Data from this phylogenetic tree supported the recombination events 3/5/11/15/20/30/41/60/67/69/76/87/92 having similar breaking points (Fig. 6c; Additional file 3: Table S3). For events 3 and 5, CM2, CM4 and their co-minor parent CM1 formed into a branch, while their major parent SM19 and UM8 belonged to other branches (Additional file 3: Table S3; Fig. 6c). For event 11, KM1 and its minor parent KM8 formed into a branch, while its major parent BM1 belonged to other main branch (Additional file 3: Table S3; Fig. 6c). In addition, the status about recombinant sequence and its minor parent having close relationship in the phylogenetic tree is also applied to events 15/20/30/41/60/67/69/76/87/92 (Additional file 3: Table S3; Fig. 6c). These data verified the reliability of recombinant events of RNA M of TSWV. Conclusively, there are frequent recombinant events among TSWV.

## Discussion

### Molecular diversity among 5 TSWV Chinese isolates

International trade of seeds and vegetables, and the presence or importation of vector thrips have allowed for the rapid spread of TSWV and associated diseases worldwide [20, 21]. During this period of expansion, TSWV has been continuing to evolve, possibly depending on host and environmental factors. For the current study, three new isolates of TSWV were cloned from different hosts (tobacco, red pepper and green pepper) grown in Chinese fields and then compared with two previously cloned TSWV isolates from tomato and lettuce in China [16]. Among these five TSWV isolates in China, only slight variations were found in the sizes of L, M and S RNA (Additional file 1: Table S1). When 29 RNA L, 62 RNA M and 66 RNA S were compared (Additional file 1: Table S1), the range of variation was considerable but mainly in untranslated regions (Table 2). For the 5 ORFs encoded by the TSWV (−) strand or (+) strand, only RdRp and NSs vary in size (15 and 204 nt, respectively) due to mutations that introduce nonsense codons (Table 2). Currently, it is not known if these size changes affect infectivity of TSWV, or if the clones represent viable viral RNAs (there is no reverse genetic system available for TSWV). CG-1 (CL5, CM5 and CS5 RNAs), isolated from lettuce in China may be considered a new TSWV species based on molecular diversity (Table 1), and phylogenetic analyses (Fig. 2). In addition, the RdRp amino acid sequence of TSWV-YN from tomato in China differs from other 4 Chinese isolates (Table 1). In contrast, YNta, YNrp and YNgp are relative stable and are found together in one branch of the RNA L and M phylogenetic trees.

The phylogenetic trees revealed that the three RNAs of the 5 Chinese TSWV isolates do not group together in the same clades, suggesting that these viruses have undergone reassortment during their evolution, as has been reported to occur for TSWV [17, 28]. RNA L of CL1, CL2, CL3 and CL4 are related to the RNA L of isolates from South Korea, while CL5 is a new species with an unclear origin (Fig. 2a). For RNA M, the origin of CM1, CM2, CM3, and CM4 is RNA M from the USA, while the origin of CM5 is RNA M from either the USA or South Korea (Fig. 2b). For RNA S, the origin of CS1, CS2, CS3 and CS4 is RNA S from South Korea, while the origin of CS5 is RNA S from either the USA or Bulgaria (Fig. 2c).

### High levels of recombination events have occurred among TSWV isolates worldwide

Besides reassortment of fragments L, M and S in different TSWV isolates, many recombination events were detectable in nearly all RNA L, M and S examined, differing from a previous report using fewer isolates that suggested only a low level of recombination events have occurred [17]. There are currently two major hypotheses concerning the contribution of recombination to the evolution of modern RNA viruses. One suggests that recombination is a major evolutionary mechanism for single-stranded RNA viruses and a minor mechanism for the evolution of multipartite RNA viruses [29, 30], which is supported by reports on *Soybean mosaic virus* and CMV [31–33]. The second hypothesis is that both recombination and reassortment are important evolutionary mechanisms for multipartite RNA viruses, which is supported by studies of *Brome mosaic virus* [34]. Our study provides additional support for the second hypothesis, although previous studies have shown that homologous recombination seems to be very rare or even absent in most negative-sense RNA viruses [35, 36].

For all three RNAs of TSWV isolates, recombination events were mainly detected in the 5′ half of the RNA. The reason for this preference is not clear. One possibility is that RdRp firstly bound RNA template, synthesized complementary strands and then had chance to facilitate recombination among different complementary strands. For bi-cistronic RNA M and S, the vast majority of recombination spanned only the ORF located at 5′, suggesting that recombination preferred to occur at ORF-coding region instead of untranslated regions and different ORFs are undergoing separate evolution. In addition, recombination events involving short fragments in the 3′ terminal region were connected with 5′ region recombination events in all three RNAs, which may cause by closed structure of TSWV RNAs resulting from the long base-pairing between 5′ and 3′ terminal sequences (5′-GAGCAAUUGUGUCA------UGACACAAUUGCUCU-3′) .

The similar recombination events in different RNA L, M and S of TSWV imply a connection between recombination and the phylogenetic tree. Isolates with similar or identical recombination breakpoints are grouped together. For example, for RNA L, three isolates (KL12, KL17, IL1) have recombination events located at positions 5155–5629 and ~8912-367 (Additional file 2: Table S2, Fig. 3), forming a cluster in the phylogenetic tree of RNA L (Fig. 2a). The second characteristic is that isolates in which no recombination was detected are usually clustered in separate groups or branches. For example, CL5 in which no recombination was detected belonged to a separate group from the other 28 isolates (Fig. 2a).

## Conclusions

Whole-genome sequences of three new TSWV isolates from different hosts in China were determined. Based on molecular diversity on 29 RNA L, 62 RNA M and 66 RNA S of TSWV, it is suggested that the entire TSWV genome, especially the M and S RNAs, have undergone extreme variations in genomic size that mainly involve the A-U rich intergenic region (IGR). Phylogenetic analyses on TSWV isolates worldwide revealed evidence for frequent reassortments. In addition, all five Chinese TSWV isolates can be divided into two groups with different origins based on molecular diversity and phylogenetic analysis. Significant numbers of recombination events were detected among TSWV isolates worldwide with apparent regional preference and showed inherent connection with phylogenetic trees. These results suggest that recombination could be an important mechanism in the evolution of multipartite RNA viruses.

## Methods

### Whole-genome cloning and sequencing of three new TSWV isolates from China

Tobacco, red pepper and green pepper plants with typical symptoms of TSWV infection were collected from Luxi county, Honghe city (Yunnan province) in China in 2013 (specific permission was not required). Three new TSWV isolates (YNta, YNrp and YNgp) were identified following cloning and sequencing. In brief, total RNA was extracted from leaves of putatively infected tobacco, red pepper or green pepper using TRIzol Reagent (TransGen). For cDNA synthesis, total RNA was combined with dNTPs and primers corresponding to the 3′ ends of L, M or S RNAs (Additional file 5: Table S5). After 5 min incubation at 65 °C, 5 units of PrimeScript reverse transcriptase and 5× buffer (Takara) was added and incubation continues at 42 °C for 1.5 h. Following cDNA synthesis, PCR amplification was performed using *LA Taq* DNA polymerase and pairs of primers corresponding to internal and terminal regions of L, M or S RNAs (Additional file 5: Table S5). Three fragments were amplified for L (positions 1–2722, 2512–5927 and 5906–8913), two fragments were amplified for M (positions 1–3401 and 2215–4772), and full-length cDNA was amplified for S. All PCR products were cloned into pMD18-T (Takara) and sequenced using universal vector primers and specific primers designed for TSWV components (Additional file 5: Table S5). At least three clones for each PCR product were sequenced to avoid experimental errors.

### Sequence assembly, construction of phylogenetic trees and recombination analysis

Sequence assembly was accomplished using DNAMAN, which was also used to analyze nucleotide and amino acids sequences among TSWV isolates. After sequence assembly, complete sequences of L, M and S for the three new TSWV isolates were deposited into GenBank with accession numbers from KM657114 to KM657122 (http://www.ncbi.nlm.nih.gov/nucleotide/).

The phylogenetic tree was constructed using the MEGA 5.0 software package [37] based on methods of neighbor-joining and Kimura 2-parameter. Bootstrap resampling (1000 replicates) was used to ensure reliability of individual nodes in the phylogenetic tree. Values lower than 70 % were hidden. Searches for recombination events employed six methods including RDP, GENECONV, Bootscan, Maxchi, Chimaera and SiScan, implemented in RDP4 [38], with likely parental isolates and recombination break points determined using default settings. Recombination events were noted if supported by at least three different methods (P-values <1.0 × 10^−6^). Sequences of additional full-length TSWV isolates were obtained from http://www.ncbi.nlm.nih.gov/nucleotide/ (See Additional file 1: Table S1 for detailed information).

## Additional files

Additional file 1: Table S1. TSWV isolates used in this study. TSWV isolates from different countries use different abbreviations as followed, China (CL, CM and CS), South Korea (KL, KM and KS), Japan (JL, JM, JS), USA (UL, UM, US), Brazil (BL, BM, BS), Italy (IL, IM IS), Australia (AM), Spain (SM, SS), Germany (GS), Bulgaria (BuS). CS: Complementary sequence; N/A: Not analyzed. (DOCX 35 kb) Additional file 2: Table S2. Summary of recombination events in different full-length TSWV L isolates using RDP4. NS: not significant. (DOCX 24 kb) Additional file 3: Table S3. Summary of recombination events in different full-length TSWV L isolates using RDP4. NS: not significant. (DOCX 31 kb) Additional file 4: Table S4 Summary of recombination events in different full-length TSWV L isolates using RDP4. NS: not significant. (DOCX 33 kb) Additional file 5: Table S5. Primers used in this study. Primers were designed based on previously reported TSWV sequences. W: A/T, M: A/C, R: G/A, Y: C/T. (DOCX 15 kb)
