# Supplementary material for: Identification of three new isolates of Tomato spotted wilt virus from different hosts in China: molecular diversity, phylogenetic and recombination analyses
Source: Virol J. 2016 Jan 14;13:8. doi: 10.1186/s12985-015-0457-3 (PMC4712509; doi:10.1186/s12985-015-0457-3)
Supplement: Additional file 1: Table S1. — TSWV isolates used in this study. TSWV isolates from different countries use different abbreviations as followed, China (CL, CM and CS), South Korea (KL, KM and KS), Japan (JL, JM, JS), USA (UL, UM, US), Brazil (BL, BM, BS), Italy (IL, IM IS), Australia (AM), Spain (SM, SS), Germany (GS), Bulgaria (BuS). CS: Complementary sequence; N/A: Not analyzed. (DOCX 35 kb) [file 12985_2015_457_MOESM1_ESM.docx]

Table S1. Detailed information for TSWV isolates used in this study

| Index | Genome RNA | Name of isolate | Region | Host | Accession No. | Size (nt) | ORF1  (nt) | ORF2  (nt) | 5’UTR  (nt) | 3’UTR  (nt) | IGR  (nt) |
| --- | --- | --- | --- | --- | --- | --- | --- | --- | --- | --- | --- |
| CL1 | L | YNta | China | Tobacco | KM657121 | 8913 | 8640 | None | 33 | 240 | None |
| CL2 | L | YNrp | China | Red pepper | KM657120 | 8913 | 8640 | None | 33 | 240 | None |
| CL3 | L | YNgp | China | Green pepper | KM657122 | 8913 | 8640 | None | 33 | 240 | None |
| CL4 | L | YN | China | Tomato | JF960237 | 8910 | 8637 | None | 33 | 240 | None |
| CL5 | L | CG-1 | China | *Lactuca sativa* | JN664254 | 8917 | 8640 | None | 34 | 243 | None |
| KL1 | L | NJ-JN | South Korea | Tomato | HM581934 | 8913 | 8640 | None | 33 | 240 | None |
| KL2 | L | CY-CN | South Korea | Pepper | HM581937 | 8914 | 8640 | None | 33 | 241 | None |
| KL3 | L | CY-CN | South Korea | Pepper | HM581940 | 8914 | 8640 | None | 33 | 241 | None |
| KL4 | L | TSWV-4 | South Korea | Pepper | KC261947 | 8913 | 8640 | None | 33 | 240 | None |
| KL5 | L | TSWV-5 | South Korea | *Stellaria aquatica* | KC261950 | 8913 | 8640 | None | 33 | 240 | None |
| KL6 | L | TSWV-6 | South Korea | *Stellaria media* | KC261953 | 8913 | 8640 | None | 33 | 240 | None |
| KL7 | L | TSWV-7 | South Korea | Pepper | KC261956 | 8913 | 8640 | None | 33 | 240 | None |
| KL8 | L | TSWV-8 | South Korea | *Lactuca indica* | KC261959 | 8913 | 8640 | None | 33 | 240 | None |
| KL10 | L | TSWV-10 | South Korea | *Stellaria aquatica* | KC261962 | 8913 | 8640 | None | 33 | 240 | None |
| KL12 | L | TSWV-12 | South Korea | Lettuce | KC261965 | 8914 | 8640 | None | 33 | 241 | None |
| KL16 | L | TSWV-16 | South Korea | Tomato | KC261968 | 8913 | 8640 | None | 33 | 240 | None |
| KL17 | L | TSWV-17 | South Korea | *Stellaria media* | KC261971 | 8914 | 8640 | None | 33 | 241 | None |
| KL18 | L | TSWV-18 | South Korea | Chrysanthemum | KC261974 | 8914 | 8640 | None | 33 | 241 | None |
| KL19 | L | LS3 | South Korea | *Leonurus sibiricus* | KM076651 | 8913 | 8640 | None | 33 | 240 | None |
| KL20 | L | Unknown | South Korea | Unknown | AB190813 | 8917 | 8643 | None | 33 | 241 | None |
| JL1 | L | Unknown | Japan | Tomato | AB198742-CS | 8913 | 8640 | None | 33 | 240 | None |
| UL1 | L | Unknown | USA | Unknown | HW047039 | 8897 | 8628 | None | 33 | 236 | None |
| UL2 | L | Unknown | USA | Unknown | HW047040 | 8897 | 8628 | None | 33 | 236 | None |
| UL3 | L | Unknown | USA | Unknown | HW047041 | 8917 | 8643 | None | 33 | 241 | None |
| UL4 | L | Unknown | USA | Unknown | HW047042-CS | 8913 | 8640 | None | 33 | 240 | None |
| BL1 | L | BR-01 | Brazil | Tomato | NC002052 | 8897 | 8628 | None | 33 | 236 | None |
| BL2 | L | Unknown | Brazil | Unknown | BD172070-CS | 8897 | N/A | None | N/A | N/A | None |
| IL1 | L | p202/3WT | Italy | Pepper | KJ575619-CS | 8914 | 8640 | None | 33 | 241 | None |
| IL2 | L | p105 | Italy | Pepper | KJ575620-CS | 8914 | 8640 | None | 33 | 241 | None |
| CM1 | M | YNta | China | Tobacco | KM657118 | 4772 | 909 | 3408 | 100 | 84 | 271 |
| CM2 | M | YNrp | China | Red pepper | KM657117 | 4771 | 909 | 3408 | 100 | 84 | 270 |
| CM3 | M | YNgp | China | Green pepper | KM657119 | 4772 | 909 | 3408 | 100 | 84 | 271 |
| CM4 | M | YN | China | Tomato | JF960236 | 4773 | 909 | 3408 | 100 | 84 | 272 |
| CM5 | M | CG-1 | China | *Lactuca sativa* | JN664253 | 4767 | 909 | 3408 | 100 | 84 | 266 |
| KM1 | M | NJ-JN | South Korea | Tomato | HM581935 | 4783 | 909 | 3408 | 100 | 84 | 282 |
| KM2 | M | CY-CN | South Korea | Pepper | HM581938 | 4768 | 909 | 3408 | 100 | 84 | 267 |
| KM3 | M | CY-CN | South Korea | Pepper | HM581941 | 4768 | 909 | 3408 | 100 | 84 | 267 |
| KM4 | M | TSWV-4 | South Korea | Pepper | KC261948 | 4781 | 909 | 3408 | 100 | 84 | 280 |
| KM5 | M | TSWV-5 | South Korea | *Stellaria aquatica* | KC261951 | 4792 | 909 | 3408 | 100 | 84 | 291 |
| KM6 | M | TSWV-6 | South Korea | *Stellaria media* | KC261954 | 4786 | 909 | 3408 | 100 | 84 | 285 |
| KM7 | M | TSWV-7 | South Korea | Pepper | KC261957 | 4785 | 909 | 3408 | 100 | 84 | 284 |
| KM8 | M | TSWV-8 | South Korea | *Lactuca indica* | KC261960 | 4787 | 909 | 3408 | 100 | 84 | 286 |
| KM10 | M | TSWV-10 | South Korea | *Stellaria aquatica* | KC261963 | 4791 | 909 | 3408 | 100 | 84 | 290 |
| KM12 | M | TSWV-12 | South Korea | Lettuce | KC261966 | 4829 | 909 | 3408 | 100 | 84 | 328 |
| KM16 | M | TSWV-16 | South Korea | Tomato | KC261969 | 4788 | 909 | 3408 | 100 | 84 | 287 |
| KM17 | M | TSWV-17 | South Korea | *Stellaria media* | KC261972 | 4828 | 909 | 3408 | 100 | 84 | 327 |
| KM18 | M | TSWV-18 | South Korea | Chrysanthemum | KC261975 | 4770 | 909 | 3408 | 100 | 84 | 269 |
| KM19 | M | LS3 | South Korea | *Leonurus sibiricus* | KM076652 | 4787 | 909 | 3408 | 100 | 84 | 286 |
| KM20 | M | Unknown | South Korea | Unknown | AB190818 | 4768 | 909 | 3408 | 100 | 85 | 266 |
| JM1 | M | Unknown | Japan | Tomato | AB010996 | 4756 | 909 | 3408 | 100 | 85 | 254 |
| UM1 | M | CA-3 | USA | Chrysanthemum | AY744481 | 4768 | 909 | 3408 | 100 | 84 | 267 |
| UM2 | M | CA-4 | USA | Chrysanthemum | AY744482 | 4767 | 909 | 3408 | 100 | 84 | 266 |
| UM3 | M | CA-5 | USA | Chrysanthemum | AY744483 | 4767 | 909 | 3408 | 100 | 84 | 266 |
| UM4 | M | CA-6 | USA | Chrysanthemum | AY744484 | 4764 | 909 | 3408 | 100 | 84 | 263 |
| UM5 | M | CA-7 | USA | Dahlia | AY744485 | 4766 | 909 | 3408 | 100 | 84 | 265 |
| UM6 | M | NC-3 | USA | Dahlia | AY744486 | 4827 | 909 | 3408 | 100 | 84 | 326 |
| UM7 | M | NC-4 | USA | Tobacco | AY744487 | 4773 | 909 | 3408 | 100 | 84 | 272 |
| UM8 | M | NC-5 | USA | Tobacco | AY744488 | 4787 | 909 | 3408 | 100 | 98 | 272 |
| UM9 | M | NC-6 | USA | Pepper | AY744489 | 4773 | 909 | 3408 | 100 | 84 | 272 |
| UM10 | M | NC-7 | USA | Tobacco | AY744490 | 4774 | 909 | 3408 | 100 | 85 | 272 |
| UM11 | M | NC-8 | USA | Tomato | AY744491 | 4774 | 909 | 3408 | 100 | 84 | 273 |
| UM12 | M | D | USA | Unknown | AF208497 | 4829 | 909 | 3408 | 100 | 84 | 328 |
| UM13 | M | Regular2A | USA | Unknown | AF208498 | 4769 | 909 | 3408 | 100 | 84 | 268 |
| UM14 | M | T | USA | *Emilia sonchifolia* | AY870389 | 4774 | 909 | 3408 | 100 | 84 | 273 |
| UM15 | M | M | USA | *Emilia sonchifolia* | AY870390 | 4763 | 909 | 3408 | 100 | 84 | 262 |
| BM1 | M | BR-01 | Brazil | Tomato | NC002050 | 4821 | 909 | 3408 | 100 | 84 | 320 |
| AM1 | M | D-191 | Australia | Tomato | HM015516 | 4824 | 909 | 3408 | 100 | 84 | 323 |
| SM1 | M | SPAIN-1 | Spain | Tomato | AY744492 | 4782 | 909 | 3408 | 100 | 84 | 281 |
| SM2 | M | SPAIN-2 | Spain | Tomato | AY744493 | 4785 | 909 | 3408 | 100 | 84 | 284 |
| SM3 | M | ALPA | Spain | Pepper | HQ537114 | 4782 | 909 | 3408 | 100 | 84 | 281 |
| SM4 | M | GRAU | Spain | *Solanum lycopersicum* | FM163370 | 4791 | 909 | 3408 | 100 | 84 | 290 |
| SM5 | M | GA-1L | Spain | *Solanum lycopersicum* | FM163371 | 4790 | 909 | 3408 | 100 | 84 | 289 |
| SM6 | M | ZO | Spain | *Solanum lycopersicum* | FM163372 | 4753 | 909 | 3408 | 100 | 84 | 252 |
| SM7 | M | LL-N.05 | Spain | *Solanum lycopersicum* | FM163373 | 4752 | 909 | 3408 | 100 | 84 | 251 |
| SM8 | M | Ab1NL2 | Spain | *Solanum lycopersicum* | HM015510 | 4784 | 909 | 3408 | 100 | 84 | 283 |
| SM9 | M | Cr1NL2 | Spain | *Solanum lycopersicum* | HM015511 | 4827 | 909 | 3408 | 100 | 84 | 326 |
| SM10 | M | Da1NL2 | Spain | *Solanum lycopersicum* | HM015512 | 4830 | 909 | 3408 | 100 | 84 | 329 |
| SM11 | M | Gr1NL2 | Spain | *Solanum lycopersicum* | HM015513 | 4781 | 909 | 3408 | 99 | 84 | 281 |
| SM12 | M | Mon1NL2 | Spain | *Solanum lycopersicum* | HM015514 | 4785 | 909 | 3408 | 100 | 84 | 284 |
| SM13 | M | Ag1TL3 | Spain | *Solanum lycopersicum* | HM015515 | 4828 | 909 | 3408 | 99 | 84 | 328 |
| SM14 | M | Ber1TL3 | Spain | *Solanum lycopersicum* | HM015517 | 4825 | 909 | 3408 | 100 | 84 | 324 |
| SM15 | M | Llo2TL3 | Spain | *Solanum lycopersicum* | HM015518 | 4787 | 909 | 3408 | 100 | 84 | 286 |
| SM16 | M | Oller1TL3 | Spain | *Solanum lycopersicum* | HM015519 | 4825 | 909 | 3408 | 100 | 84 | 324 |
| SM17 | M | Pujol1TL3 | Spain | *Solanum lycopersicum* | HM015520 | 4825 | 909 | 3408 | 100 | 84 | 324 |
| SM18 | M | Sala1TL3 | Spain | *Solanum lycopersicum* | HM015521 | 4826 | 909 | 3408 | 100 | 84 | 325 |
| SM19 | M | ViTL3 | Spain | *Solanum lycopersicum* | HM015522 | 4787 | 909 | 3408 | 100 | 84 | 286 |
| SM20 | M | Gr5TL1 | Spain | *Solanum lycopersicum* | HM015523 | 4791 | 909 | 3408 | 100 | 84 | 290 |
| SM21 | M | Rib1TL1 | Spain | *Solanum lycopersicum* | HM015524 | 4786 | 909 | 3408 | 100 | 84 | 285 |
| IM1 | M | p202/3WT | Italy | Pepper | HQ830188 | 4824 | 909 | 3408 | 100 | 84 | 323 |
| IM2 | M | p202/3RB | Italy | Pepper | HQ830185 | 4824 | 909 | 3408 | 100 | 84 | 323 |
| IM3 | M | p105 | Italy | Pepper | KJ575621 | 4766 | 909 | 3408 | 100 | 84 | 265 |
| CS1 | S | YNta | China | Tobacco | KM657115 | 2970 | 1404 | 777 | 88 | 151 | 550 |
| CS2 | S | YNrp | China | Red pepper | KM657114 | 2968 | 1404 | 777 | 88 | 151 | 548 |
| CS3 | S | YNgp | China | Green pepper | KM657116 | 2971 | 1404 | 777 | 88 | 151 | 551 |
| CS4 | S | YN | China | Tomato | JF960235 | 2970 | 1404 | 777 | 88 | 151 | 550 |
| CS5 | S | CG-1 | China | *Lactuca sativa* | JN664252 | 2920 | 1401 | 777 | 86 | 153 | 503 |
| CS6 | S | KM-T | China | Tomato | HQ402595 | 2971 | 1404 | 777 | 88 | 151 | 551 |
| KS1 | S | NJ-JN | South Korea | Tomato | HM581936 | 2968 | 1404 | 777 | 88 | 151 | 548 |
| KS2 | S | CY-CN | South Korea | Pepper | HM581939 | 3013 | 1404 | 777 | 88 | 151 | 593 |
| KS3 | S | CY-CN | South Korea | Pepper | HM581942 | 3013 | 1404 | 777 | 88 | 151 | 593 |
| KS4 | S | TSWV-4 | South Korea | Pepper | KC261949 | 2971 | 1404 | 777 | 88 | 151 | 551 |
| KS5 | S | TSWV-5 | South Korea | *Stellaria aquatica* | KC261952 | 2975 | 1404 | 777 | 88 | 151 | 555 |
| KS6 | S | TSWV-6 | South Korea | *Stellaria media* | KC261955 | 2969 | 1404 | 777 | 88 | 151 | 549 |
| KS7 | S | TSWV-7 | South Korea | Pepper | KC261958 | 2967 | 1404 | 777 | 88 | 151 | 547 |
| KS8 | S | TSWV-8 | South Korea | *Lactuca indica* | KC261961 | 2977 | 1404 | 777 | 88 | 151 | 557 |
| KS10 | S | TSWV-10 | South Korea | *Stellaria aquatica* | KC261964 | 2975 | 1404 | 777 | 88 | 151 | 555 |
| KS12 | S | TSWV-12 | South Korea | Lettuce | KC261967 | 2961 | 1404 | 777 | 88 | 151 | 541 |
| KS16 | S | TSWV-16 | South Korea | Tomato | KC261970 | 2973 | 1404 | 777 | 88 | 151 | 553 |
| KS17 | S | TSWV-17 | South Korea | *Stellaria media* | KC261973 | 2961 | 1404 | 777 | 88 | 151 | 541 |
| KS18 | S | TSWV-18 | South Korea | Chrysanthemum | KC261976 | 3020 | 1404 | 777 | 88 | 151 | 600 |
| KS19 | S | LS3 | South Korea | *Leonurus sibiricus* | KM076653 | 2973 | 1404 | 777 | 88 | 151 | 553 |
| KS20 | S | Unknown | South Korea | Unknown | AB190819 | 2991 | 1404 | 777 | 88 | 151 | 571 |
| JS1 | S | Ordinary strain | Japan | Tomato | AB088385 | 2999 | 1404 | 777 | 88 | 152 | 578 |
| US1 | S | TSWV-10 | USA | Unknown | AF020659 | 3017 | 1404 | 777 | 88 | 151 | 597 |
| US2 | S | TSWV-D | USA | Unknown | AF020660 | 2955 | 1404 | 777 | 88 | 151 | 535 |
| US3 | S | CA-1 | USA | Aster | AY744468 | 2927 | 1404 | 777 | 88 | 151 | 507 |
| US4 | S | CA-2 | USA | Buttercup | AY744469 | 2926 | 1404 | 777 | 88 | 151 | 506 |
| US5 | S | CA-3 | USA | Chrysanthemum | AY744470 | 2921 | 1404 | 777 | 88 | 151 | 501 |
| US6 | S | CA-4 | USA | Chrysanthemum | AY744471 | 2921 | 1404 | 777 | 88 | 151 | 501 |
| US7 | S | CA-5 | USA | Chrysanthemum | AY744472 | 2921 | 1404 | 777 | 88 | 151 | 501 |
| US8 | S | CA-6 | USA | Chrysanthemum | AY744473 | 2920 | 1404 | 777 | 88 | 151 | 500 |
| US9 | S | CA-7 | USA | Dahlia | AY744474 | 2927 | 1404 | 777 | 88 | 151 | 507 |
| US10 | S | CO | USA | Falso lulo | AY744475 | 2923 | 1404 | 777 | 88 | 151 | 503 |
| US11 | S | NC-1 | USA | Dahlia | AY744476 | 2959 | 1404 | 777 | 88 | 151 | 539 |
| US12 | S | NC-2 | USA | Peanut | AY744477 | 3021 | 1404 | 777 | 88 | 151 | 601 |
| US13 | S | NC-3 | USA | Dahlia | AY744478 | 2954 | 1404 | 777 | 88 | 151 | 534 |
| US14 | S | M | USA | *Emilia sonchifolia* | AY870391 | 3047 | 1404 | 777 | 88 | 153 | 625 |
| US15 | S | T | USA | *Emilia sonchifolia* | AY870392 | 3016 | 1404 | 777 | 88 | 153 | 594 |
| US16 | S | TSWV-B | USA | Unknown | L12048 | 3049 | 1404 | 777 | 87 | 151 | 630 |
| BS1 | S | BR-01 | Brazil | Tomato | NC002051 | 2916 | 1395 | 777 | 88 | 153 | 505 |
| BS2 | S | Br20RB | Brazil | Pepper | DQ915947 | 2926 | 1404 | 777 | 87 | 153 | 504 |
| BS3 | S | Br20 | Brazil | Pepper | DQ915948 | 2926 | 1404 | 777 | 87 | 153 | 504 |
| SS1 | S | SPAIN-1 | Spain | Tomato | AY744479 | 2922 | 1404 | 777 | 88 | 151 | 502 |
| SS2 | S | SPAIN-2 | Spain | Tomato | AY744480 | 2923 | 1404 | 777 | 88 | 151 | 503 |
| SS3 | S | VE430 | Spain | Pepper | DQ376184 | 2922 | 1404 | 777 | 88 | 151 | 502 |
| SS4 | S | VE427 | Spain | Pepper | DQ376185 | 2922 | 1404 | 777 | 88 | 151 | 502 |
| IS1 | S | p202/3RB | Italy | Pepper | HQ830186 | 2962 | 1332 | 777 | 88 | 151 | 614 |
| IS2 | S | p202/3WT | Italy | Pepper | HQ830187 | 2963 | 1404 | 777 | 88 | 151 | 543 |
| IS3 | S | p105-RB-Mar | Italy | Pepper | HQ839729 | 2926 | 1332 | 777 | 88 | 151 | 578 |
| IS4 | S | p105-RB-MaxI | Italy | Pepper | HQ839730 | 2927 | 972 | 777 | 88 | 151 | 939 |
| IS5 | S | p105-RB-MaxII | Italy | Pepper | HQ839731 | 2927 | 1404 | 777 | 88 | 151 | 507 |
| IS6 | S | P105-1 | Italy | Pepper | DQ376177 | 2927 | 1404 | 777 | 88 | 151 | 507 |
| IS7 | S | P105 | Italy | Pepper | DQ376178 | 2927 | 1404 | 777 | 88 | 151 | 507 |
| IS8 | S | P166 | Italy | Pepper | DQ376179 | 2925 | 1404 | 777 | 88 | 151 | 505 |
| IS9 | S | P267 | Italy | Pepper | DQ376180 | 2927 | 1404 | 777 | 88 | 151 | 507 |
| IS10 | S | P272 | Italy | Pepper | DQ376181 | 2934 | 1404 | 777 | 88 | 151 | 514 |
| IS11 | S | P105-43.14 | Italy | Pepper | DQ376182 | 2927 | 1404 | 777 | 88 | 151 | 507 |
| IS12 | S | P105-44.7 | Italy | Pepper | DQ376183 | 2926 | 1404 | 777 | 88 | 151 | 506 |
| IS13 | S | p202 | Italy | Pepper (open field) | DQ398945 | 2961 | 1200 | 777 | 88 | 151 | 745 |
| IS14 | S | p105/2006RB | Italy | Pepper | DQ915946 | 2927 | 1404 | 777 | 88 | 151 | 507 |
| IS15 | S | p170 | Italy | Pepper (open field) | DQ431237 | 2954 | 1404 | 777 | 88 | 151 | 534 |
| IS16 | S | P170RB | Italy | Pepper (glasshouse) | DQ431238 | 2930 | 717 | 777 | 88 | 151 | 1197 |
| GS1 | S | LE98/527 | Germany | *Lysimachia sp.* | AJ418781 | 2965 | 1401 | 777 | 88 | 151 | 548 |
| BuS1 | S | GD98 | Bulgaria | Oriental tobacco | AJ418780 | 3006 | 1401 | 777 | 90 | 151 | 587 |
| BuS2 | S | DH37 | Bulgaria | Tomato greenhouse | AJ418779 | 2948 | 1404 | 777 | 88 | 151 | 528 |
| BuS3 | S | 10HK96 | Bulgaria | Virginia tobacco | AJ418778 | 3364 | 1404 | 777 | 88 | 152 | 943 |
| BuS4 | S | BS97 | Bulgaria | Virginia tobacco | AJ418777 | 2958 | 1404 | 777 | 88 | 151 | 538 |

Note:

1. TSWVs from different countries used different abbreviation for index as followed, China (CL, CM and CS), South Korea (KL, KM and KS), Japan (JL, JM, JS), USA (UL, UM, US), Brazil (BL, BM, BS), Italy (IL, IM IS), Australia (AM), Spain (SM, SS), Germany (GS), Bulgaria (BuS)
2. ORF: Open reading frame; UTR: Untranslated region; IGR: Internal gene region; nt: nucleotide; CS: Complementary sequence; N/A: Not analyzed
