# Supplementary material for: Identification of three new isolates of Tomato spotted wilt virus from different hosts in China: molecular diversity, phylogenetic and recombination analyses
Source: Virol J. 2016 Jan 14;13:8. doi: 10.1186/s12985-015-0457-3 (PMC4712509; doi:10.1186/s12985-015-0457-3)
Supplement: Additional file 2: Table S2. — Summary of recombination events in different full-length TSWV L isolates using RDP4. NS: not significant. (DOCX 24 kb) [file 12985_2015_457_MOESM2_ESM.docx]

Table S2. Summary of recombination events in different full-length TSWV L fragments identified by RDP4 program.

|  |  | **Breakpoint position in recombinant sequence** | | **Parental sequence (s)** | | **P-Value for the six detection methods in RDP4** | | | | | |
| --- | --- | --- | --- | --- | --- | --- | --- | --- | --- | --- | --- |
| **Event number** | **Recombinant Sequence(s)** | **Begin** | **End** | **Minor** | **Major** | **RDP** | **GENECONV** | **BootScan** | **MaxChi** | **Chimaera** | **SiScan** |
| 1 | CL1 | 27 | 367 | Unknown(UL3) | UL4 | 1.84E-22 | 1.391E-13 | 9.231E-23 | NS | 8.212E-03 | 2.889E-03 |
| 2 | CL1 | 27 | 608 | UL2 | IL2 | 1.71E-21 | 1.68E-16 | 4.42E-26 | 3.84E-08 | NS | 3.78E-07 |
| 3 | CL1 | 1319 | 2011 | KL4 | UL3 | 1.64E-70 | 2.75E-41 | 4.48E-69 | 2.50E-13 | 4.91E-14 | 1.14E-20 |
| 4 | CL2 | 27 | 367 | Unknown(UL3) | UL4 | 1.84E-22 | 1.391E-13 | 9.231E-23 | NS | 8.212E-03 | 2.889E-03 |
| 5 | CL2 | 27 | 609 | UL2 | IL2 | 1.71E-21 | 1.68E-16 | 4.42E-26 | 3.84E-08 | NS | 3.78E-07 |
| 6 | CL2 | 8722 | 4138 | KL4 | UL3 | 1.64E-70 | 2.75E-41 | 4.48E-69 | 2.50E-13 | 4.91E-14 | 1.14E-20 |
| 7 | CL3 | 27 | 367 | Unknown(UL3) | UL4 | 1.84E-22 | 1.391E-13 | 9.231E-23 | NS | 8.212E-03 | 2.889E-03 |
| 8 | CL3 | 116 | 462 | UL2 | IL2 | 1.71E-21 | 1.68E-16 | 4.42E-26 | 3.84E-08 | NS | 3.78E-07 |
| 9 | CL3 | 8722 | 4138 | KL4 | UL3 | 1.64E-70 | 2.75E-41 | 4.48E-69 | 2.50E-13 | 4.91E-14 | 1.14E-20 |
| 10 | CL4 | 25 | 622 | CL2 | Unknown(JL1) | 1.27E-44 | 1.22E-39 | 2.17E-42 | 4.93E-09 | 3.09E-09 | 1.73E-10 |
| 11 | CL4 | 623 | 2834 | CL2 | Unknown(JL1) | 1.96E-74 | 2.62E-98 | 3.92E-62 | 1.13E-21 | 3.68E-02 | 7.89E-29 |
| 12 | CL4 | 643 | 2817 | CL3 | Unknown(KL19) | 1.19E-106 | 3.19E-84 | 3.54E-88 | 2.31E-20 | 2.55E-20 | 1.15E-26 |
| 13 | CL4 | 2863 | 4676 | CL1 | Unknown(KL2) | 3.74E-84 | 6.44E-81 | 3.09E-87 | 2.42E-18 | 8.58E-19 | 1.49E-27 |
| 14 | CL4 | 2863 | 4690 | JL1 | Unknown(CL1) | 1.35E-85 | 2.93E-83 | 1.82E-88 | 1.40E-19 | 2.65E-20 | 6.64E-28 |
| 15 | CL4 | 2922 | 4690 | CL1 | Unknown(JL1) | 1.91E-92 | 1.88E-89 | 2.48E-95 | 1.02E-19 | NS | 1.02E-28 |
| 16 | CL4 | 4781 | 6936 | CL3 | Unknown(KL2) | 8.00E-91 | 2.687E-90 | 1.187E-93 | 1.71E-24 | 6.63-26 | 4.31E-36 |
| 17 | KL1 | 27 | 367 | Unknown(UL3) | JL1 | 4.53E-24 | 5.53E-15 | 1.35E-23 | 3.57E-04 | 2.31E-03 | 1.46E-03 |
| 18 | KL1 | 27 | 606 | UL2 | IL2 | 1.71E-21 | 1.68E-16 | 4.42E-26 | 3.84E-08 | NS | 3.78E-07 |
| 19 | KL1 | 5154 | 5634 | Unknown(KL18) | KL19 | 9.46E-07 | 7.49E-06 | 3.44E-08 | 3.74E-08 | 3.55E-09 | 1.15E-14 |
| 20 | KL2 | 27 | 2833 | UL3 | KL16 | 8.63E-125 | 1.27E-69 | 8.29E-122 | 7.61E-21 | 9.57E-22 | 1.67E-26 |
| 21 | KL2 | 8912 | 2523 | UL3 | KL4 | 1.64E-70 | 2.75E-41 | 4.48E-69 | 2.50E-13 | 4.91E-14 | 1.14E-20 |
| 22 | KL3 | 27 | 2833 | UL3 | KL16 | 8.63E-125 | 1.27E-69 | 8.29E-122 | 7.61E-21 | 9.57E-22 | 1.67E-26 |
| 23 | KL3 | 8912 | 2523 | UL3 | KL4 | 1.64E-70 | 2.75E-41 | 4.48E-69 | 2.50E-13 | 4.91E-14 | 1.14E-20 |
| 24 | KL4 | 27 | 367 | Unknown(UL3) | JL1 | 4.53E-24 | 5.53E-15 | 1.35E-23 | 3.57E-04 | 2.31E-03 | 1.46E-03 |
| 25 | KL4 | 27 | 606 | UL2 | IL2 | 1.71E-21 | 1.68E-16 | 4.42E-26 | 3.84E-08 | NS | 3.78E-07 |
| 26 | KL4 | 5154 | 5640 | Unknown(KL18) | KL19 | 9.46E-07 | 7.49E-06 | 3.44E-08 | 3.74E-08 | 3.55E-09 | 1.15E-14 |
| 27 | KL5 | 27 | 367 | Unknown(UL3) | JL1 | 4.53E-24 | 5.53E-15 | 1.35E-23 | 3.57E-04 | 2.31E-03 | 1.46E-03 |
| 28 | KL5 | 27 | 606 | UL2 | IL2 | 1.71E-21 | 1.68E-16 | 4.42E-26 | 3.84E-08 | NS | 3.78E-07 |
| 29 | KL5 | 5154 | 5634 | Unknown(KL18) | KL19 | 9.46E-07 | 7.49E-06 | 3.44E-08 | 3.74E-08 | 3.55E-09 | 1.15E-14 |
| 30 | KL6 | 27 | 367 | Unknown(UL3) | JL1 | 4.53E-24 | 5.53E-15 | 1.35E-23 | 3.57E-04 | 2.31E-03 | 1.46E-03 |
| 31 | KL6 | 27 | 606 | UL2 | IL2 | 1.71E-21 | 1.68E-16 | 4.42E-26 | 3.84E-08 | NS | 3.78E-07 |
| 32 | KL6 | 5154 | 5634 | Unknown(KL18) | KL19 | 9.46E-07 | 7.49E-06 | 3.44E-08 | 3.74E-08 | 3.55E-09 | 1.15E-14 |
| 33 | KL7 | 27 | 367 | Unknown(UL3) | JL1 | 4.53E-24 | 5.53E-15 | 1.35E-23 | 3.57E-04 | 2.31E-03 | 1.46E-03 |
| 34 | KL7 | 27 | 606 | UL2 | IL2 | 1.71E-21 | 1.68E-16 | 4.42E-26 | 3.84E-08 | NS | 3.78E-07 |
| 35 | KL7 | 5154 | 5634 | Unknown(KL18) | KL19 | 9.46E-07 | 7.49E-06 | 3.44E-08 | 3.74E-08 | 3.55E-09 | 1.15E-14 |
| 36 | KL8 | 27 | 367 | Unknown(UL3) | JL1 | 4.53E-24 | 5.53E-15 | 1.35E-23 | 3.57E-04 | 2.31E-03 | 1.46E-03 |
| 37 | KL8 | 27 | 606 | UL2 | IL2 | 1.71E-21 | 1.68E-16 | 4.42E-26 | 3.84E-08 | NS | 3.78E-07 |
| 38 | KL8 | 5154 | 5634 | Unknown(KL18) | KL19 | 9.46E-07 | 7.49E-06 | 3.44E-08 | 3.74E-08 | 3.55E-09 | 1.15E-14 |
| 39 | KL10 | 27 | 367 | Unknown(UL3) | JL1 | 4.53E-24 | 5.53E-15 | 1.35E-23 | 3.57E-04 | 2.31E-03 | 1.46E-03 |
| 40 | KL10 | 27 | 606 | UL2 | IL2 | 1.71E-21 | 1.68E-16 | 4.42E-26 | 3.84E-08 | NS | 3.78E-07 |
| 41 | KL10 | 5154 | 5634 | Unknown(KL18) | KL19 | 9.46E-07 | 7.49E-06 | 3.44E-08 | 3.74E-08 | 3.55E-09 | 1.15E-14 |
| 42 | KL12 | 8912 | 367 | Unknown(UL3) | JL1 | 4.53E-24 | 5.53E-15 | 1.35E-23 | 3.57E-04 | 2.31E-03 | 1.46E-03 |
| 43 | KL12 | 71 | 606 | UL2 | IL2 | 1.71E-21 | 1.68E-16 | 4.42E-26 | 3.84E-08 | NS | 3.78E-07 |
| 44 | KL12 | 5155 | 5629 | Unknown(KL18) | KL19 | 9.46E-07 | 7.49E-06 | 3.44E-08 | 3.74E-08 | 3.55E-09 | 1.15E-14 |
| 45 | KL16 | 27 | 367 | Unknown(UL3) | JL1 | 4.53E-24 | 5.53E-15 | 1.35E-23 | 3.57E-04 | 2.31E-03 | 1.46E-03 |
| 46 | KL16 | 27 | 606 | UL2 | IL2 | 1.71E-21 | 1.68E-16 | 4.42E-26 | 3.84E-08 | NS | 3.78E-07 |
| 47 | KL16 | 5154 | 5634 | Unknown(KL18) | KL19 | 9.46E-07 | 7.49E-06 | 3.44E-08 | 3.74E-08 | 3.55E-09 | 1.15E-14 |
| 48 | KL17 | 64 | 606 | UL2 | IL2 | 1.71E-21 | 1.68E-16 | 4.42E-26 | 3.84E-08 | NS | 3.78E-07 |
| 49 | KL17 | 8913 | 367 | Unknown(UL3) | JL1 | 4.53E-24 | 5.53E-15 | 1.35E-23 | 3.57E-04 | 2.31E-03 | 1.46E-03 |
| 50 | KL17 | 5155 | 5629 | Unknown(KL18) | KL19 | 9.46E-07 | 7.49E-06 | 3.44E-08 | 3.74E-08 | 3.55E-09 | 1.15E-14 |
| 51 | KL18 | 27 | 2833 | UL3 | KL16 | 8.63E-125 | 1.27E-69 | 8.29E-122 | 7.61E-21 | 9.57E-22 | 1.67E-26 |
| 52 | KL18 | 8912 | 2523 | UL3 | KL4 | 1.64E-70 | 2.75E-41 | 4.48E-69 | 2.50E-13 | 4.91E-14 | 1.14E-20 |
| 53 | KL19 | 3 | 367 | Unknown(UL3) | JL1 | 4.53E-24 | 5.53E-15 | 1.35E-23 | 3.57E-04 | 2.31E-03 | 1.46E-03 |
| 54 | KL19 | 27 | 606 | UL2 | IL2 | 1.71E-21 | 1.68E-16 | 4.42E-26 | 3.84E-08 | NS | 3.78E-07 |
| 55 | KL19 | 8128 | 1379 | KL4 | UL3 | 1.64E-70 | 2.75E-41 | 4.48E-69 | 2.50E-13 | 4.91E-14 | 1.14E-20 |
| 56 | KL19 | 1795 | 1990 | KL2 | KL16 | 3.38E-09 | 4.53E-08 | 3.27E-09 | 1.18E-02 | 1.15E-02 | 1.70E-03 |
| 57 | KL19 | 2199 | 2600 | KL2 | KL17 | 1.02E-02 | 3.19E-07 | 3.35E-09 | NS | NS | 6.38E-08 |
| 58 | KL19 | 2895 | 3279 | KL2 | KL10 | 5.56E-09 | 9.06E-08 | 5.59E-09 | 4.14E-03 | 4.03E-03 | 2.84E-03 |
| 59 | KL19 | 5154 | 5634 | KL18 | KL16 | 5.37E-13 | 1.70E-11 | 4.70E-13 | 1.55E-08 | 7.58E-09 | 2.01E-09 |
| 60 | KL19 | 5154 | 7248 | UL4 | KL1 | 1.41E-06 | NS | 1.17E-06 | 8.11E-12 | 6.64E-10 | 4.36E-09 |
| 61 | KL19 | 6308 | 6716 | KL18 | KL16 | 1.46E-08 | 3.65E-07 | 1.13E-07 | 4.50E-03 | 2.45E-03 | 2.08E-02 |
| 62 | KL19 | 6910 | 7248 | KL2 | KL1 | 7.28E-09 | 2.30E-07 | 6.97E-09 | 1.07E-05 | 2.22E-05 | 1.37E-04 |
| 63 | KL20 | 123 | 4161 | KL18 | Unknown(KL10) | 2.67E-122 | 5.84E-76 | 1.10E-63 | 6.92E-37 | 1.02E-21 | 2.11E-78 |
| 64 | KL20 | 2944 | 4143 | KL3 | Unknown(CL3) | 1.34E-64 | 2.17E-47 | 1.22E-57 | 7.8E-14 | 1.55E-14 | 3.39E-19 |
| 65 | KL20 | 2856 | 4162 | KL3 | Unknown(KL1) | 1.03E-74 | 1.62E-54 | 1.74E-69 | 1.91E-14 | 7.08E-15 | 3.00E-17 |
| 66 | JL1 | 27 | 2822 | UL3 | KL16 | 8.63E-125 | 1.27E-69 | 8.29E-122 | 7.61E-21 | 9.57E-22 | 1.67E-26 |
| 67 | JL1 | 8746 | 2865 | UL3 | KL4 | 1.64E-70 | 2.75E-41 | 4.48E-69 | 2.50E-13 | 4.91E-14 | 1.14E-20 |
| 68 | UL1 | 19 | 622 | KL1 | Unknown(UL4) | 2.73E-19 | 3.31E-10 | NS | 1.27E-09 | 2.58E-08 | NS |
| 69 | UL1 | 617 | 2865 | IL1 | Unknown(IL2) | 3.18E-16 | 1.67E-04 | 6.78E-42 | 4.02E-10 | NS | 6.73E-74 |
| 70 | UL1 | 674 | 1338 | IL2 | Unknown(KL12) | 1.03E-20 | 2.05E-05 | 1.04E-09 | 4.58E-10 | 1.27E-07 | 6.59E-04 |
| 71 | UL2 | 19 | 622 | KL1 | Unknown(UL4) | 2.73E-19 | 3.31E-10 | NS | 1.27E-09 | 2.58E-08 | NS |
| 72 | UL2 | 674 | 1338 | IL2 | Unknown(KL12) | 1.03E-20 | 2.05E-05 | 1.04E-09 | 4.58E-10 | 1.27E-07 | 6.59E-04 |
| 73 | UL2 | 617 | 2865 | IL1 | Unknown(IL2) | 3.18E-16 | 1.67E-04 | 6.78E-42 | 4.02E-10 | NS | 6.73E-74 |
| 74 | UL2 | 8896 | 617 | KL10 | Unknown(JL1 | 1.47E-28 | 2.91E-19 | NS | 1.76E-09 | 5.11E-08 | NS |
| 75 | UL3 | 2944 | 4143 | KL3 | Unknown(CL3) | 1.34E-64 | 2.17E-47 | 1.22E-57 | 7.8E-14 | 1.55E-14 | 3.39E-19 |
| 76 | UL3 | 3214 | 4161 | KL3 | Unknown(KL1) | 1.03E-74 | 1.62E-54 | 1.74E-69 | 1.91E-14 | 7.08E-15 | 3.00E-17 |
| 77 | UL3 | 8726 | 4162 | KL18 | Unknown(KL10) | 2.67E-122 | 5.84E-76 | 1.10E-63 | 6.92E-37 | 1.02E-21 | 2.11E-78 |
| 78 | UL4 | 27 | 2790 | UL3 | KL4 | 1.64E-70 | 2.75E-41 | 4.48E-69 | 2.50E-13 | 4.91E-14 | 1.14E-20 |
| 79 | UL4 | 27 | 2833 | UL3 | KL16 | 8.63E-125 | 1.27E-69 | 8.29E-122 | 7.61E-21 | 9.57E-22 | 1.67E-26 |
| 80 | BL1 | 19 | 622 | KL1 | Unknown(UL4) | 2.73E-19 | 3.31E-10 | NS | 1.27E-09 | 2.58E-08 | NS |
| 81 | BL1 | 78 | 1003 | KL10 | Unknown(JL1 | 1.47E-28 | 2.91E-19 | NS | 1.76E-09 | 5.11E-08 | NS |
| 82 | BL1 | 674 | 1407 | IL2 | Unknown(KL12) | 1.03E-20 | 2.05E-05 | 1.04E-09 | 4.58E-10 | 1.27E-07 | 6.59E-04 |
| 83 | BL1 | 617 | 1407 | IL1 | Unknown(IL2) | 3.18E-16 | 1.67E-04 | 6.78E-42 | 4.02E-10 | NS | 6.73E-74 |
| 84 | IL1 | 27 | 617 | UL2 | IL2 | 1.71E-21 | 1.68E-16 | 4.42E-26 | 3.84E-08 | NS | 3.78E-07 |
| 85 | IL1 | 5155 | 5629 | Unknown(KL18) | KL19 | 9.46E-07 | 7.49E-06 | 3.44E-08 | 3.74E-08 | 3.55E-09 | 1.15E-14 |
| 86 | IL1 | 8904 | 367 | Unknown(UL3) | JL1 | 4.53E-24 | 5.53E-15 | 1.35E-23 | 3.57E-04 | 2.31E-03 | 1.46E-03 |
| 87 | IL2 | 68 | 2579 | UL3 | KL16 | 8.63E-125 | 1.27E-69 | 8.29E-122 | 7.61E-21 | 9.57E-22 | 1.67E-26 |
| 88 | IL2 | 8912 | 2771 | UL3 | KL4 | 1.64E-70 | 2.75E-41 | 4.48E-69 | 2.50E-13 | 4.91E-14 | 1.14E-20 |

NS: not significant
