# Supplementary material for: Identification of three new isolates of Tomato spotted wilt virus from different hosts in China: molecular diversity, phylogenetic and recombination analyses
Source: Virol J. 2016 Jan 14;13:8. doi: 10.1186/s12985-015-0457-3 (PMC4712509; doi:10.1186/s12985-015-0457-3)
Supplement: Additional file 3: Table S3. — Summary of recombination events in different full-length TSWV L isolates using RDP4. NS: not significant. (DOCX 31 kb) [file 12985_2015_457_MOESM3_ESM.docx]

Table S3. Summary of recombination events in different full-length TSWV M fragments identified by RDP4 program.

|  |  | **Breakpoint position in recombinant sequence** | | **Parental sequence (s)** | | **P-Value for the six detection methods in RDP4** | | | | | |
| --- | --- | --- | --- | --- | --- | --- | --- | --- | --- | --- | --- |
| **Event number** | **Recombinant Sequence(s)** | **Begin** | **End** | **Minor** | **Major** | **RDP** | **GENECONV** | **BootScan** | **MaxChi** | **Chimaera** | **SiScan** |
| 1 | CM1 | 754 | 1208 | CM2 | CM3 | 2.46E-85 | 1.04E-81 | 6.01E-85 | 3.27E-12 | 3.24E-12 | 5.15E-16 |
| 2 | CM2 | 48 | 753 | CM1 | Unknown(IM2) | 8.88E-05 | 1.01E-12 | 2.64E-15 | NS | NS | 2.34E-09 |
| 3 | CM2 | 60 | 1208 | CM1 | Unknown(SM19) | 2.86E-28 | 3.90E-55 | 6.86E-30 | 6.82E-16 | 2.10E-10 | 1.70E-07 |
| 4 | CM2 | 754 | 1208 | CM1 | Unknown(CM3) | 2.46E-85 | 1.04E-81 | 6.01E-85 | 3.27E-12 | 3.24E-12 | 1.82E-16 |
| 5 | CM4 | 49 | 1210 | CM1 | UM8 | 4.37E-133 | 2.43E-125 | 3.23E-133 | 8.16E-22 | 5.29E-06 | 5.73E-18 |
| 6 | CM4 | 4770 | 1230 | CM2 | Unknown(UM14) | 1.97E-32 | 1.47E-62 | 2.27E-65 | 5.36E-17 | 1.05E-11 | 8.51E-15 |
| 7 | CM5 | 584 | 1016 | Unknown(UM4) | KM20 | 2.70E-41 | 6.01E-43 | 4.90E-49 | 2.37E-09 | 1.72E-05 | 1.50E-07 |
| 8 | CM5 | 4743 | 1110 | KM20 | UM13 | 9.60E-30 | NS | 5.56E-15 | 1.33E-18 | 1.28E-12 | NS |
| 9 | CM5 | 4750 | 1125 | KM20 | UM5 | 2.17E-30 | NS | 5.55E-28 | 2.14E-19 | 9.49E-13 | 2.61E-16 |
| 10 | KM1 | 18 | 1159 | KM8 | Unknown(SM18) | 1.44E-24 | 1.21E-39 | 5.97E-20 | 8.62E-10 | 1.85E-09 | 2.38E-15 |
| 11 | KM1 | 27 | 1183 | KM8 | BM1 | 1.91E-42 | 9.91E-83 | 2.91E-63 | 1.48E-11 | 3.02E-14 | 2.73E-19 |
| 12 | KM1 | 4732 | 1218 | SM19 | Unknown(SM14) | 1.00E-27 | 2.53E-41 | 3.04E-55 | 6.95E-16 | 8.35E-12 | NS |
| 13 | KM2 | 336 | 1059 | UM7 | IM1 | 1.37E-03 | 3.90E-07 | 1.03E-05 | 1.12E-07 | 6.12E-04 | 1.06E-12 |
| 14 | KM4 | 4753 | 1096 | SM12 | AM1 | 2.88E-10 | 1.10E-20 | 7.55E-17 | 1.22E-07 | 4.16E-05 | 1.22E-08 |
| 15 | KM5 | 19 | 1182 | KM16 | Unknown(BM1) | 4.65E-47 | 7.37E-103 | 4.28E-77 | 5.41E-05 | 4.46E-16 | 9.49E-28 |
| 16 | KM5 | 4745 | 1186 | KM7 | KM17 | 1.15E-30 | 7.46E-50 | 5.54E-40 | 8.90E-15 | 1.05E-09 | 2.01E-15 |
| 17 | KM6 | 57 | 1144 | Unknown(SM5) | SM21 | 4.45E-35 | 2.67E-30 | 1.19E-33 | 4.71E-04 | 9.57E-05 | NS |
| 18 | KM6 | 4757 | 1194 | SM2 | Unknown(AM1) | 1.10E-10 | 7.02E-14 | 6.18E-19 | 4.29E-09 | 3.97E-06 | 1.32E-08 |
| 19 | KM7 | 29 | 1104 | KM6 | AM1 | 2.09E-11 | 8.84E-22 | 1.48E-22 | 1.12E-07 | 1.49E-04 | 3.09E-05 |
| 20 | KM7 | 56 | 1164 | KM10 | SM12 | 1.66E-28 | 7.68E-18 | 1.23E-19 | 3.13E-06 | 1.09E-03 | NS |
| 21 | KM7 | 63 | 1655 | KM1 | SM7 | 1.24E-09 | 3.50E-28 | 6.87E-16 | 1.95E-03 | 3.72E-06 | 8.31E-07 |
| 22 | KM7 | 4765 | 1080 | KM4 | KM20 | 1.76E-04 | 9.74E-10 | 3.46E-12 | 5.73E-07 | 5.19E-04 | 2.11E-06 |
| 23 | KM7 | 4770 | 1192 | SM15 | SM2 | 7.49E-23 | 1.81E-12 | 2.00E-08 | 8.46E-11 | 2.65E-05 | NS |
| 24 | KM8 | 58 | 1119 | Unknown(SM5) | SM15 | 2.27E-31 | 8.07E-23 | 4.94E-31 | 2.58E-04 | 5.41E-05 | 6.80E-05 |
| 25 | KM8 | 1127 | 1222 | Unknown(UM12) | SM19 | 1.21E-24 | 7.94E-23 | 9.47E-16 | 1.63E-02 | NS | 1.19E-03 |
| 26 | KM8 | 4751 | 1194 | SM2 | Unknown(AM1) | 1.10E-10 | 7.02E-14 | 6.18E-19 | 4.29E-09 | 3.97E-06 | 1.32E-08 |
| 27 | KM10 | 36 | 1164 | KM19 | UM6 | 2.07E-22 | 8.35E-41 | 1.18E-15 | NS | NS | NS |
| 28 | KM10 | 4744 | 1238 | SM18 | Unknown(SM8) | 5.40E-08 | 1.23E-12 | 1.86E-16 | NS | NS | 3.19E-10 |
| 29 | KM12 | 7 | 106 | Unknown(UM1) | UM7 | 3.07E-10 | 1.63E-21 | 3.43E-25 | 6.96E-08 | 2.00E-07 | 9.03E-09 |
| 30 | KM12 | 41 | 1208 | KM17 | UM12 | 1.28E-135 | 5.36E-139 | 3.91E-141 | 1.81E-21 | 1.79E-21 | 2.50E-24 |
| 31 | KM12 | 4763 | 1210 | KM17 | SM5 | 2.08E-29 | 5.05E-49 | 3.50E-43 | 1.83E-20 | 5.443E-07 | 1.89E-11 |
| 32 | KM16 | 29 | 1186 | KM7 | SM9 | 4.41E-29 | 2.43E-51 | 2.05E-34 | 2.96E-02 | 1.95E-12 | 4.91E-17 |
| 33 | KM16 | 63 | 1704 | KM1 | SM7 | 1.24E-09 | 3.50E-28 | 6.87E-16 | 1.95E-08 | 3.72E-06 | 8.31E-07 |
| 34 | KM16 | 4750 | 1220 | KM10 | UM6 | 1.18E-39 | 1.83E-75 | 5.48E-51 | 1.96E-05 | 9.42E-10 | 1.77E-19 |
| 35 | KM17 | 120 | 1120 | Unknown(SM1) | SM15 | 4.74E-23 | 2.04E-26 | 1.11E-20 | 6.67E-08 | 5.30E-08 | 4.18E-15 |
| 36 | KM17 | 4718 | 1107 | Unknown(KM10) | KM19 | 1.46E-28 | 2.49E-43 | 1.93E-47 | NS | NS | 4.67E-16 |
| 37 | KM17 | 4769 | 1206 | SM9 | Unknown(UM6) | NS | NS | NS | 1.52E-14 | 9.44E-13 | 5.89E-10 |
| 38 | KM19 | 1 | 1096 | Unknown(SM5) | SM15 | 2.85E-38 | 7.01E-30 | 2.26E-38 | 1.43E-05 | 5.44E-05 | NS |
| 39 | KM19 | 443 | 1095 | KM6 | SM19 | 8.42E-29 | 1.88E-16 | 6.05E-29 | 9.54E-03 | 3.85E-03 | 6.79E-04 |
| 40 | KM20 | 4742 | 1094 | Unknown(UM4) | UM2 | 2.44E-48 | 4.81E-39 | 2.76E-46 | 3.70E-10 | 7.54E-07 | 7.89E-09 |
| 41 | UM2 | 3 | 1070 | UM4 | CM5 | 6.76E-26 | 3.05E-18 | 4.04E-26 | 2.12E-03 | 9.85E-03 | 6.02E-04 |
| 42 | UM2 | 4744 | 1212 | Unknown(UM13) | KM20 | 9.60E-30 | NS | 5.56E-15 | 1.33E-18 | 1.28E-12 | NS |
| 43 | UM2 | 4758 | 1015 | UM4 | KM20 | 2.70E-41 | 6.06E-43 | 4.90E-49 | 2.37E-09 | 1.72E-05 | 1.50E-07 |
| 44 | UM3 | 33 | 993 | UM4 | CM5 | 6.76E-26 | 3.05E-18 | 4.04E-26 | 2.12E-03 | 9.85E-03 | 6.02E-04 |
| 45 | UM3 | 4742 | 1212 | Unknown(UM5) | KM20 | 2.17E-30 | NS | 5.55E-28 | 2.14E-19 | 9.49E-13 | 2.61E-16 |
| 46 | UM4 | 4742 | 1086 | UM3 | Unknown(KM20) | 2.79E-84 | 1.02E-88 | 4.52E-83 | 2.08E-13 | 7.30E-15 | 3.34E-11 |
| 47 | UM5 | 4742 | 1212 | KM20 | Unknown(UM2) | 1.86E-32 | NS | NS | 1.09E-22 | 1.09E-13 | 3.36E-16 |
| 48 | UM6 | 55 | 1074 | Unknown(UM15) | UM7 | 4.33E-19 | 3.19E-39 | 2.24E-26 | 2.03E-13 | 6.86E-08 | 6.45E-10 |
| 49 | UM6 | 120 | 1113 | Unknown(SM5) | SM1 | 3.89E-17 | 9.65E-33 | 2.40E-28 | 4.60E-09 | 1.57E-05 | 4.30E-13 |
| 50 | UM6 | 4770 | 1126 | Unknown(KM8) | KM16 | 4.56E-24 | 1.64E-46 | 2.22E-34 | 5.65E=13 | 3.21E-08 | 2.60E-16 |
| 51 | UM7 | 49 | 1199 | Unknown(CM1) | CM4 | 3.47E-128 | 3.98E-121 | 3.61E-129 | 1.81E-21 | 5.21E-16 | 5.37E-16 |
| 52 | UM7 | 61 | 1248 | Unknown(CM2) | CM4 | 8.80E-127 | 3.84E-117 | 1.63E-127 | 2.91E-21 | 4.18E-17 | 1.18E-20 |
| 53 | UM8 | 49 | 1210 | Unknown(CM1) | CM4 | 3.47E-128 | 3.98E-121 | 3.61E-129 | 1.81E-21 | 5.21E-16 | 5.37E-16 |
| 54 | UM8 | 61 | 1248 | Unknown(CM2) | CM4 | 8.80E-127 | 3.84E-117 | 1.63E-127 | 2.91E-21 | 4.18E-17 | 1.18E-20 |
| 55 | UM8 | 672 | 1198 | UM14 | UM10 | 2.99E-77 | 6.12E-74 | 1.74E-77 | 2.63E-11 | 1.25E-11 | 2.59E-05 |
| 56 | UM9 | 27 | 1199 | Unknown(CM1) | CM4 | 3.47E-128 | 3.98E-121 | 3.61E-129 | 1.81E-21 | 5.21E-16 | 5.37E-16 |
| 57 | UM9 | 61 | 1229 | Unknown(CM2) | CM4 | 8.80E-127 | 3.84E-117 | 1.63E-127 | 2.91E-21 | 4.18E-17 | 1.18E-20 |
| 58 | UM10 | 61 | 1248 | Unknown(CM2) | CM4 | 8.80E-127 | 3.84E-117 | 1.63E-127 | 2.91E-21 | 4.18E-17 | 1.18E-20 |
| 59 | UM10 | 4770 | 1229 | Unknown(CM2) | CM4 | 2.78E-124 | 5.81E-114 | 1.09E-72 | 1.84E-21 | 2.78E-21 | 9.45E-23 |
| 60 | UM11 | 55 | 1191 | UM13 | SM14 | 1.17E-25 | 3.21E-31 | 1.71E-33 | 6.17E-14 | 4.25E-06 | 2.21E-09 |
| 61 | UM11 | 214 | 1197 | UM8 | Unknown(UM10) | 2.86E-71 | 2.17E-74 | 3.42E--32 | 6.84E-11 | 1.57E-11 | 1.11E-04 |
| 62 | UM11 | 4759 | 1218 | UM13 | UM6 | 9.67E-21 | 6.01E-28 | 9.70E-36 | 8.23E-12 | 1.31E-04 | 2.97E-09 |
| 63 | UM12 | 7 | 869 | Unknown(UM1) | UM7 | 3.07E-10 | 1.63E-21 | 3.43E-25 | 6.96E-08 | 2.00E-07 | 9.03E-09 |
| 64 | UM12 | 42 | 1222 | KM17 | KM12 | 1.28E-136 | 5.36E-139 | 1.24E-135 | 4.07E-21 | 1.57E-21 | 1.17E-26 |
| 65 | UM12 | 102 | 1260 | Unknown(KM20) | IM2 | 8.17E-19 | 6.19E-34 | 2.99E-35 | NS | NS | NS |
| 66 | UM12 | 4770 | 1115 | Unknown(SM15) | SM5 | 4.07E-19 | 8.18E-30 | 9.91E-16 | 4.84E-12 | 6.77E-06 | 2.03E-16 |
| 67 | UM13 | 54 | 1227 | UM8 | Unknown(UM6) | 2.38E-16 | 4.19E-26 | 9.75E-39 | 2.33E-14 | 6.75E-10 | 1.95E-08 |
| 68 | UM13 | 124 | 1192 | UM14 | SM18 | 2.97E-21 | 7.01E-26 | 6.59E-32 | 7.00E-14 | 3.86E-06 | 1.95E-13 |
| 69 | UM14 | 67 | 1194 | UM13 | Unknown(CM2) | 7.75E-12 | NS | 3.39E-09 | 1.13E-07 | 1.26E-06 | 3.79E-05 |
| 70 | UM14 | 672 | 1198 | UM8 | Unknown(UM10) | 2.86E-74 | 2.17E-74 | 3.42E-32 | 6.84E-11 | 1.57E-11 | 1.11E-04 |
| 71 | UM14 | 4759 | 1180 | UM13 | UM6 | 9.67E-21 | 6.01E-28 | 9.70E-36 | 8.23E-12 | 1.31E-04 | 2.97E-09 |
| 72 | UM15 | 54 | 1074 | UM7 | SM18 | 7.07E-20 | 2.70E-40 | 3.91E-31 | 1.85E-13 | 2.95E-08 | 7.63E-09 |
| 73 | UM15 | 124 | 1140 | UM9 | Unknown(KM12) | 3.57E-21 | 2.10E-37 | 4.15E-40 | 3.93E-12 | 7.71E-06 | 5.07E-17 |
| 74 | SM1 | 124 | 1099 | SM21 | Unknown(SM14) | 9.85E-30 | 1.67E-52 | 4.63E-37 | 3.85E-09 | 4.02E-05 | 4.03E-13 |
| 75 | SM1 | 4766 | 1224 | SM15 | KM20 | 5.53E-12 | 7.33E-27 | 3.24E-19 | 1.03E-08 | 4.66E-05 | 1.21E-03 |
| 76 | SM2 | 5 | 1190 | Unknown(KM10) | KM7 | 4.47E-59 | 1.02E-13 | 1.90E-50 | 1.34E-10 | 1.59E-09 | 4.44E-08 |
| 77 | SM2 | 818 | 1190 | Unknown(KM16) | KM7 | 7.46E-55 | 8.94E-14 | 3.68E-51 | 5.52E-09 | 6.46E-08 | 1.58E-05 |
| 78 | SM2 | 4756 | 817 | KM4 | Unknown(KM20) | 1.76E-04 | 9.74E-10 | 3.46E-12 | 5.73E-07 | 5.19E-04 | 2.11E-06 |
| 79 | SM3 | 53 | 1118 | SM21 | Unknown(KM20) | 2.14E-13 | 3.95E-24 | 1.82E-17 | 1.39E-10 | 8.64E-07 | 9.59E-14 |
| 80 | SM3 | 4762 | 1117 | SM12 | AM1 | 2.88E-10 | 1.10E-20 | 7.55E-17 | 1.22E-07 | 4.16E-05 | 1.22E-08 |
| 81 | SM4 | 37 | 359 | KM19 | Unknown(UM6) | 2.07E-22 | 8.35E-41 | 1.18E-15 | NS | NS | NS |
| 82 | SM4 | 57 | 1225 | Unknown(KM16) | KM10 | 1.72E-99 | 4.56E-94 | 5.56E-58 | 3.43E-16 | 5.15E-16 | 2.38E-19 |
| 83 | SM4 | 63 | 1355 | KM1 | SM7 | 1.24E-09 | 350E-28 | 6.87E-16 | 1.95E-08 | 3.72E-06 | 8.31E-07 |
| 84 | SM4 | 4662 | 1101 | SM21 | Unknown(SM14) | 9.85E-30 | 1.67E-52 | 4.63E-37 | 3.85E-09 | 4.02E-05 | 4.03E-13 |
| 85 | SM4 | 4769 | 1214 | KM16 | Unknown(SM9) | 7.93E-25 | 1.40E-47 | 1.49E-33 | 4.76E-13 | 4.56E-06 | 5.41E-16 |
| 86 | SM5 | 29 | 1101 | SM21 | Unknown(SM14) | 9.85E-30 | 1.67E-52 | 4.63E-37 | 3.85E-09 | 4.02E-05 | 4.03E-13 |
| 87 | SM5 | 131 | 1229 | SM1 | Unknown(UM4) | 1.20E-11 | 2.53E-26 | 5.52E-28 | 3.67E-07 | NS | 1.84E-11 |
| 88 | SM5 | 4767 | 1099 | SM15 | SM14 | 3.65E-23 | 3.03E-51 | 1.14E-31 | 1.45E-15 | 1.70E-09 | 3.05E-11 |
| 89 | SM6 | 246 | 1066 | UM7 | Unknown(IM1) | 1.34E-03 | 3.90E-07 | 1.03E-05 | 1.12E-07 | 6.12E-04 | 1.06E-12 |
| 90 | SM7 | 37 | 1094 | UM1 | UM6 | 6.17E-16 | 4.16E-28 | 1.97E-24 | 1.04E-10 | 1.91E-05 | NS |
| 91 | SM7 | 4668 | 1212 | UM5 | Unknown(UM12) | 2.83E-06 | 7.59E-10 | 3.47E-10 | NS | NS | 2.59E-06 |
| 92 | SM8 | 19 | 1269 | KM8 | BM1 | 1.91E-42 | 9.91E-83 | 2.91E-63 | 1.48E-11 | 3.02E-14 | 6.30E-18 |
| 93 | SM9 | 37 | 1022 | Unknown(SM15) | SM1 | 2.33E-14 | 6.62E-29 | 1.75E-24 | 8.53E-11 | 1.37E-06 | 4.07E-11 |
| 94 | SM9 | 4765 | 1038 | Unknown(SM5) | SM1 | 3.89E-17 | 9.65E-33 | 2.40E-28 | 4.60E-09 | 1.57E-05 | 4.30E-13 |
| 95 | SM10 | 37 | 1148 | Unknown(KM1) | KM10 | 2.27E-21 | 4.90E-45 | 2.87E-29 | 2.58E-13 | 4.11E-10 | 2.11E-19 |
| 96 | SM10 | 4769 | 1132 | Unknown(KM8) | KM10 | 2.16E-26 | 5.55E-43 | 2.08E-45 | 1.05E-13 | 5.63E-08 | 1.40E-12 |
| 97 | SM12 | 8 | 1192 | SM1 | SM2 | 1.67E-28 | 3.12E-11 | 1.88E-08 | 5.39E-06 | 1.25E-05 | NS |
| 98 | SM12 | 4652 | 1186 | KM10 | Unknown(SM7) | 4.85E-08 | NS | 1.78E-12 | 1.90E-04 | NS | 3.78E-10 |
| 99 | SM13 | 7 | 2626 | UM6 | Unknown(SM9) | NS | NS | NS | 1.52E-14 | 9.44E-13 | 5.89E-10 |
| 100 | SM14 | 29 | 1373 | Unknown(KM10) | KM6 | 3.77E-26 | 6.47E-57 | 8.36E-41 | 9.57E-15 | 2.68E-07 | 1.60E-17 |
| 101 | SM14 | 825 | 1087 | Unknown(KM17) | SM17 | 1.74E-49 | 9.07E-44 | 1.63E-03 | 3.52E-06 | 4.47E-07 | 6.01E-09 |
| 102 | SM14 | 4742 | 784 | Unknown(SM12) | SM5 | 9.73E-11 | 3.54E-20 | 4.73E-19 | 1.31E-12 | 5.64E-08 | 1.26E-07 |
| 103 | SM14 | 4758 | 1100 | Unknown(SM15) | SM21 | 4.53E-26 | 3.59E-47 | 2.19E-32 | 2.87E-02 | 1.21E-10 | 5.86E-10 |
| 104 | SM15 | 150 | 1118 | SM3 | Unknown(UM8) | 3.02E-06 | 4.48E-13 | 9.12E-11 | 9.99E-07 | 9.94E-06 | 1.07E-03 |
| 105 | SM15 | 4764 | 1118 | SM12 | AM1 | 2.88E-10 | 1.10E-20 | 7.55E-17 | 1.22E-07 | 4.16E-05 | 1.22E-08 |
| 106 | SM15 | 4771 | 1175 | SM5 | KM19 | 3.76E-42 | 4.17E-26 | 3.00E-35 | 5.15E-06 | 2.78E-07 | 2.89E-03 |
| 107 | SM16 | 29 | 1104 | Unknown(KM7) | KM6 | 3.51E-25 | 2.23E-51 | 4.93E-39 | 7.45E-14 | 5.35E-09 | 2.78E-17 |
| 108 | SM16 | 37 | 1107 | Unknown(KM10) | KM19 | 3.35E-25 | 3.87E-47 | 6.15E-50 | 5.44E-16 | 1.03E-10 | 8.83E-21 |
| 109 | SM16 | 825 | 1087 | Unknown(KM17) | SM17 | 1.74E-49 | 9.07E-44 | 1.63E-03 | 3.52E-06 | 4.47E-07 | 6.01E-09 |
| 110 | SM16 | 4758 | 1109 | Unknown(SM15) | SM21 | 4.53E-26 | 3.59E-47 | 2.19E-32 | 2.87E-02 | 1.21E-10 | 5.86E-10 |
| 111 | SM17 | 29 | 1104 | Unknown(KM7) | KM6 | 3.51E-25 | 2.23E-51 | 4.93E-39 | 7.45E-14 | 5.35E-09 | 2.78E-17 |
| 112 | SM17 | 37 | 1107 | Unknown(KM10) | KM19 | 3.35E-25 | 3.87E-47 | 6.15E-50 | 5.44E-16 | 1.03E-10 | 8.83E-21 |
| 113 | SM17 | 240 | 1140 | Unknown(KM10) | KM6 | 3.77E-26 | 6.47E-57 | 8.36E-41 | 9.57E-15 | 2.68E-07 | 1.60E-17 |
| 114 | SM17 | 4758 | 1109 | Unknown(SM15) | SM21 | 4.53E-26 | 3.59E-47 | 2.19E-32 | 2.87E-02 | 1.21E-10 | 5.86E-10 |
| 115 | SM18 | 29 | 1092 | Unknown(KM6) | KM8 | 1.13E-23 | 8.13E-43 | 2.97E-32 | 2.97E-12 | 2.15E-07 | 3.05E-13 |
| 116 | SM18 | 117 | 1095 | Unknown(SM12) | SM5 | 1.14E-17 | 1.05E-37 | 7.80E-29 | 1.18E-10 | 2.63E-06 | NS |
| 117 | SM18 | 388 | 1125 | Unknown(KM10) | KM8 | 9.12E-29 | 1.52E-54 | 2.10E-35 | 1.77E-13 | 2.82E-05 | 2.57E-20 |
| 118 | SM19 | 2 | 1174 | SM5 | KM19 | 3.76E-42 | 4.17E-26 | 3.00E-35 | 5.15E-06 | 2.78E-07 | 2.89E-03 |
| 119 | SM19 | 4764 | 1118 | SM12 | AM1 | 2.88E-10 | 1.10E-20 | 7.55E-17 | 1.22E-07 | 4.16E-05 | 1.22E-08 |
| 120 | SM20 | 29 | 1101 | SM21 | Unknown(SM14) | 9.85E-30 | 1.67E-52 | 4.63E-37 | 3.85E-09 | 4.02E-05 | 4.03E-13 |
| 121 | SM20 | 37 | 359 | KM19 | Unknown(UM6) | 2.07E-22 | 8.35E-41 | 1.18E-15 | NS | NS | NS |
| 122 | SM20 | 57 | 1225 | Unknown(KM16) | KM10 | 1.72E-99 | 4.56E-94 | 5.56E-58 | 3.43E-16 | 5.15E-16 | 2.38E-19 |
| 123 | SM20 | 63 | 1355 | KM1 | SM7 | 1.24E-09 | 3.50E-26 | 6.87E-16 | 1.95E-03 | 3.72-06 | 8.31E-07 |
| 124 | SM20 | 4769 | 1214 | KM16 | Unknown(SM9) | 7.93E-25 | 1.40E-47 | 1.49E-33 | 4.76E-13 | 4.56E-06 | 5.41E-16 |
| 125 | SM21 | 2 | 1155 | SM15 | Unknown(KM19) | 1.58E-35 | 2.27E-22 | 1.26E-24 | 1.54E-03 | 4.84E-05 | NS |
| 126 | SM21 | 4753 | 1194 | SM2 | Unknown(AM1) | 1.10E-10 | 7.02E-14 | 6.18E-19 | 4.29E-09 | 3.99E-06 | 1.32E-08 |
| 127 | BM1 | 27 | 975 | Unknown(IM1) | AM1 | 4.28E-14 | 4.15E-18 | 7.61E-16 | 1.50E-06 | 3.00E-17 | NS |
| 128 | BM1 | 120 | 1118 | Unknown(SM15) | SM1 | 1.07E-39 | 2.51E-80 | 2.25E-60 | 9.85E-03 | 8.40E-13 | 2.91E-17 |
| 129 | BM1 | 4756 | 1182 | Unknown(KM5) | KM16 | 4.65E-47 | 2.80E-102 | 4.28E-77 | 1.19E-04 | 2.62E-15 | 2.16E-16 |
| 130 | AM1 | 104 | 1212 | Unknown(BM1) | SM12 | 3.89E-12 | 3.16E-11 | 5.81E-14 | 4.12E-09 | 1.45E-02 | 5.24E-10 |
| 131 | AM1 | 123 | 1104 | Unknown(KM3) | JM1 | 3.89E-12 | 5.35E-13 | 8.80E-15 | 3.22E-05 | 1.45E-02 | NS |
| 132 | AM1 | 4750 | 1082 | Unknown(SM7) | KM3 | 1.80E-03 | 5.02E-19 | 2.30E-15 | 3.74E-07 | 1.28E-03 | NS |
| 133 | IM1 | 37 | 809 | Unknown(KM3) | JM1 | 3.89E-12 | 3.16E-11 | 5.81E-14 | 4.12E-09 | 1.45E-02 | 5.24E-10 |
| 134 | IM1 | 123 | 874 | Unknown(KM3) | JM1 | 3.89E-12 | 5.35E-13 | 8.80E-15 | 3.23E-05 | 1.45E-02 | NS |
| 135 | IM1 | 974 | 1251 | SM17 | Unknown(SM14) | 6.45E-55 | 3.07E-53 | 4.60E-54 | 1.33E-07 | 6.50E-09 | 1.69E-11 |
| 136 | IM1 | 4723 | 250 | KM3 | Unknown(SM7) | 1.80E-03 | 5.02E-19 | 2.30E-15 | 3.74E-07 | 1.28E-03 | NS |
| 137 | IM1 | 4762 | 1270 | SM17 | AM1 | 5.62E-152 | 1.37E-144 | 2.97E-02 | 1.19E-24 | 1.45E-25 | 1.04E-28 |
| 138 | IM2 | 37 | 809 | Unknown(KM3) | JM1 | 3.89E-12 | 3.16E-11 | 5.81E-14 | 4.12E-09 | 1.45E-02 | 5.24E-10 |
| 139 | IM2 | 123 | 874 | Unknown(KM3) | JM1 | 3.89E-12 | 5.35E-13 | 8.80E-15 | 3.23E-05 | 1.45E-02 | NS |
| 140 | IM2 | 268 | 1270 | SM17 | AM1 | 5.62E-152 | 1.37E-144 | 1.27E-148 | 6.15E-25 | 1.14E-25 | 1.04E-28 |
| 141 | IM2 | 974 | 1251 | SM17 | Unknown(SM14) | 6.45E-55 | 3.07E-53 | 4.60E-54 | 1.33E-07 | 6.50E-09 | 1.69E-11 |
| 142 | IM3 | 4743 | 1212 | KM20 | Unknown(UM2) | 1.86E-32 | NS | NS | 1.09E-22 | 1.09E-13 | 3.36E-16 |
| 143 | IM3 | 4762 | 1269 | SM17 | AM1 | 5.62E-152 | 1.37E-144 | 2.97E-02 | 1.19E-24 | 1.45E-25 | 1.04E-28 |

NS: not significant
