# Supplementary material for: Identification of three new isolates of Tomato spotted wilt virus from different hosts in China: molecular diversity, phylogenetic and recombination analyses
Source: Virol J. 2016 Jan 14;13:8. doi: 10.1186/s12985-015-0457-3 (PMC4712509; doi:10.1186/s12985-015-0457-3)
Supplement: Additional file 4: Table S4 — Summary of recombination events in different full-length TSWV L isolates using RDP4. NS: not significant. (DOCX 33 kb) [file 12985_2015_457_MOESM4_ESM.docx]

Table S4. Summary of recombination events in different full-length TSWV S fragments identified by RDP4 program.

|  |  | **Breakpoint position in recombinant sequence** | | **Parental sequence (s)** | | **P-Value for the six detection methods in RDP4** | | | | | |
| --- | --- | --- | --- | --- | --- | --- | --- | --- | --- | --- | --- |
| **Event number** | **Recombinant Sequence(s)** | **Begin** | **End** | **Minor** | **Major** | **RDP** | **GENECONV** | **BootScan** | **MaxChi** | **Chimaera** | **SiScan** |
| 1 | CS1 | 173 | 1852 | CS3 | KS17 | 2.64E-37 | 1.27E-76 | 2.12E-51 | 2.47E-06 | 1.34E-15 | 2.88E-23 |
| 2 | CS1 | 179 | 1880 | CS6 | US8 | 3.78E-33 | 1.25E-58 | 1.19E-44 | 3.60E-16 | 5.33E-15 | 1.84E-20 |
| 3 | CS2 | 449 | 1708 | CS6 | BuS3 | 1.12E-31 | 7.08E-64 | 1.83E-55 | 8.66E-04 | 1.54E-14 | 1.59E-16 |
| 4 | CS2 | 2966 | 1825 | KS4 | US14 | 9.78E-20 | 5.61E-36 | 2.84E-25 | 2.55E-09 | 3.97E-10 | 1.46E-17 |
| 5 | CS3 | 2899 | 1772 | KS19 | BuS3 | 5.98E-22 | 1.05E-41 | 5.50E-05 | 7.15E-17 | 1.01E-11 | 2.25E-11 |
| 6 | CS3 | 37 | 1798 | CS1 | US10 | 6.27E-19 | 5.49E-64 | 1.03E-54 | NS | 1.03E-10 | 5.03E-18 |
| 7 | CS4 | 22 | 1896 | CS6 | US8 | 3.78E-33 | 1.25E-58 | 1.19E-44 | 3.60E-16 | 5.33E-15 | 1.84E-20 |
| 8 | CS4 | 116 | 1792 | KS19 | US2 | 4.65E-25 | 3.27E-41 | 7.50E-19 | 9.88E-16 | 1.02E-10 | 2.73E-19 |
| 9 | CS5 | 1728 | 1781 | SS2 | Unknown(KS18) | 2.95E-20 | 6.32E-08 | NS | 8.81E-07 | 4.38E-06 | 1.18E-06 |
| 10 | CS6 | 2908 | 1772 | KS19 | BuS3 | 5.98E-22 | 1.05E-41 | 5.50E-05 | 7.15E-17 | 1.01E-11 | 2.25E-11 |
| 11 | CS6 | 69 | 1972 | CS1 | US10 | 6.27E-19 | 5.49E-64 | 1.03E-54 | NS | 1.03E-10 | 5.03E-18 |
| 12 | JS1 | 202 | 1638 | IS2 | Unknown(US5) | 4.83E-14 | 8.68E-45 | 2.14E-22 | 1.02E-11 | 2.89E-07 | 2.88E-23 |
| 13 | KS1 | 2964 | 1786 | KS19 | US15 | 3.68E-26 | 1.66E-72 | 1.85E-47 | 1.58E-06 | 2.60E-17 | 5.13E-24 |
| 14 | KS1 | 410 | 1760 | KS4 | Unknown(IS1) | 8.85E-17 | 5.47E-48 | NS | 2.63E-12 | NS | 2.72E-32 |
| 15 | KS1 | 11 | 1785 | KS19 | Unknown(US14) | 7.68E-44 | 3.71E-83 | 4.55E-55 | 6.39E-06 | 8.83E-18 | 4.68E-20 |
| 16 | KS1 | 29 | 1861 | KS19 | IS1 | 3.50E-53 | 3.80E-94 | 1.39E-77 | 2.31E-30 | 1.21E-19 | 1.36E-39 |
| 17 | KS2 | 5 | 1831 | KS18 | Unknown(BuS3) | 4.77E-36 | 2.12E-67 | 2.90E-48 | 2.17E-21 | 5.31E-15 | 1.26E-25 |
| 18 | KS3 | 5 | 1752 | KS18 | Unknown(BuS3) | 4.77E-36 | 2.12E-67 | 2.90E-48 | 2.17E-21 | 5.31E-15 | 1.26E-25 |
| 19 | KS4 | 431 | 1622 | BuS4 | Unknown(KS8) | 1.27E-16 | 3.26E-25 | 3.18E-35 | 2.38E-14 | 2.63E-09 | 8.63E-17 |
| 20 | KS4 | 908 | 1779 | CS1 | US10 | 6.27E-19 | 5.49E-64 | 1.03E-54 | NS | 1.03E-54 | 5.03E-18 |
| 21 | KS5 | 2883 | 1760 | KS8 | US5 | 1.73E-35 | 3.48E-68 | 1.06E-46 | 2.13E-06 | 1.72E-18 | 4.79E-25 |
| 22 | KS5 | 110 | 1732 | KS8 | US8 | 2.24E-37 | 9.58E-67 | 9.94E-38 | 1.00E-03 | 2.07E-15 | 3.67E-20 |
| 23 | KS5 | 8 | 1609 | KS19 | Unknown(BuS3) | 8.34E-25 | 1.49E-31 | 8.06E-36 | 1.89E-03 | 1.05E-05 | 9.12E-19 |
| 24 | KS6 | 2946 | 1813 | KS19 | CS2 | 4.13E-32 | 3.70E-41 | 2.01E-34 | 9.54E-13 | 7.08E-10 | 1.49E-10 |
| 25 | KS6 | 321 | 1883 | KS4 | Unknown(IS1) | 8.85E-17 | 5.47E-48 | NS | 2.63E-12 | NS | 2.72E-32 |
| 26 | KS7 | 109 | 1899 | CS6 | US8 | 3.78E-33 | 1.25E-58 | 1.19E-44 | 3.60E-16 | 5.33E-15 | 1.84E-20 |
| 27 | KS7 | 116 | 1758 | KS4 | US14 | 9.78E-20 | 5.61E-36 | 2.84E-25 | 2.55E-09 | 3.97E-10 | 1.46E-17 |
| 28 | KS7 | 81 | 1707 | BuS3 | KS19 | 5.06E-31 | 7.50E-65 | 3.93E-47 | 3.51E-19 | 3.23E-12 | 1.90E-25 |
| 29 | KS8 | 2965 | 1760 | KS10 | Unknown(BuS3) | 3.95E-36 | 5.50E-69 | 2.68E-54 | 1.17E-04 | 3.21E-15 | 3.50E-19 |
| 30 | KS10 | 2887 | 1782 | KS8 | US8 | 2.24E-37 | 9.58E-67 | 9.94E-38 | 1.00E-03 | 2.07E-15 | 3.67E-20 |
| 31 | KS10 | 2966 | 1609 | KS19 | BuS3 | 8.34E-25 | 1.49E-31 | 8.06E-36 | 1.89E-03 | 1.05E-05 | 9.12E-19 |
| 32 | KS12 | 2959 | 1869 | IS2 | IS13 | 1.85E-137 | 7.58E-75 | 2.79E-131 | 8.01E-43 | 2.75E-42 | 1.43E-48 |
| 33 | KS12 | 2914 | 1368 | IS1 | Unknown(BS3) | 8.86E-05 | 1.67E-15 | 3.39E-17 | 6.00E-06 | 1.86E-02 | 6.33E-10 |
| 34 | KS16 | 2914 | 1785 | KS1 | US14 | 4.56E-42 | 2.92E-79 | 8.03E-61 | 2.31E-15 | 1.34E-16 | 2.48E-20 |
| 35 | KS16 | 2813 | 1616 | BuS3 | KS5 | 9.57E-22 | 2.01E-53 | 2.63E-35 | 3.57E-07 | 2.53E-10 | 1.52E-18 |
| 36 | KS17 | 2959 | 1864 | IS2 | IS13 | 1.85E-137 | 7.58E-75 | 2.79E-131 | 8.01E-43 | 2.75E-42 | 1.43E-48 |
| 37 | KS17 | 2914 | 1368 | IS1 | Unknown(BS3) | 8.86E-05 | 1.67E-15 | 3.39E-17 | 6.00E-06 | 1.86E-02 | 6.33E-10 |
| 38 | KS17 | 59 | 1879 | IS2 | CS1 | 3.97E-21 | 1.04E-35 | 1.46E-34 | NS | NS | 2.29E-21 |
| 39 | KS18 | 2921 | 1936 | KS3 | US6 | NS | 1.71E-59 | 2.36E-44 | 2.10E-22 | 1.86E-15 | 2.12E-28 |
| 40 | KS18 | 2952 | 1752 | KS2 | BuS3 | 1.32E-36 | 5.29E-68 | 1.02E-47 | 1.88E-20 | 3.13E-11 | 9.28E-19 |
| 41 | KS19 | 2917 | 1786 | KS1 | US14 | 4.56E-42 | 2.92E-79 | 8.03E-61 | 2.31E-15 | 1.34E-16 | 2.48E-20 |
| 42 | KS19 | 2952 | 1763 | KS4 | Unknown(IS2) | 5.72E-33 | 3.71E-56 | 2.81E-04 | 4.14E-05 | 9.37E-14 | 5.78E-25 |
| 43 | KS19 | 8 | 1609 | BuS3 | KS5 | 9.57E-22 | 2.01E-53 | 2.63E-35 | 3.57E-07 | 2.53E-10 | 1.52E-18 |
| 44 | KS20 | 2952 | 1762 | KS2 | US14 | 9.70E-10 | 2.74E-11 | 4.09E-17 | NS | N | 6.56E-08 |
| 45 | KS20 | 98 | 1629 | Unknown(US1) | US12 | NS | 1.84E-34 | 3.58E-17 | 9.95E-15 | 9.15E-09 | 1.70E-17 |
| 46 | US1 | 70 | 1919 | US15 | Unknown(BuS3) | 1.30E-25 | 1.16E-39 | 2.02E-41 | 2.80E-22 | 8.48E-15 | 9.44E-23 |
| 47 | US1 | 98 | 1964 | US15 | US13 | 5.83E-28 | 2.86E-42 | 4.32E-37 | 1.09E-05 | 9.60E-17 | 2.60E-20 |
| 48 | US1 | 1166 | 1692 | US7 | Unknown(IS12) | 1.61E-08 | NS | NS | 1.38E-07 | 3.99E-06 | NS |
| 49 | US2 | 462 | 1759 | Unknown(K4) | KS7 | 5.69E-10 | 1.05E-39 | 8.50E-21 | 1.40E-09 | 7.16E-11 | 3.47E-21 |
| 50 | US2 | 2955 | 1564 | KS12 | BS3 | 1.97E-06 | 6.04E-24 | 7.44E-23 | 2.13E-09 | 1.40E-08 | 1.26E-08 |
| 51 | US3 | 2921 | 1970 | JS1 | US7 | 3.10E-11 | 4.68E-22 | 1.21E-26 | 5.21E-09 | NS | 2.58E-05 |
| 52 | US3 | 3 | 1789 | Unknown(IS12) | IS6 | 3.61E-107 | 3.28E-105 | 5.45E-09 | 4.65E-27 | 3.40E-27 | 1.29E-29 |
| 53 | US3 | 1156 | 1696 | Unknown(US10) | IS12 | 3.62E-08 | 8.20E-18 | 6.55E-22 | 8.96E-07 | 7.25E-04 | 9.08E-12 |
| 54 | US4 | 2924 | 1784 | IS6 | IS3 | 3.28E-22 | 7.34E-78 | 4.09E-141 | 4.51E-26 | 7.77E-22 | 8.60E-25 |
| 55 | US4 | 1310 | 1629 | Unknown(IS12) | US15 | 5.64E-07 | NS | 1.22E-20 | 3.93E-09 | 2.73E-06 | 3.14E-02 |
| 56 | US4 | 2926 | 1881 | US7 | Unknown(BuS2) | 2.42E-22 | 3.54E-44 | 2.86E-23 | 7.02E-12 | 1.62E-03 | 7.73E-15 |
| 57 | US5 | 2899 | 1803 | IS2 | US8 | 1.18E-33 | 4.04E-69 | 9.16E-43 | 2.55E-22 | 2.84E-13 | 6.64E-18 |
| 58 | US5 | 40 | 1520 | BuS3 | Unknown(BuS4) | 6.02E-09 | 6.43E-11 | 9.33E-11 | 2.51E-05 | 1.49E-05 | 8.59E-12 |
| 59 | US5 | 2877 | 1780 | Unknown(KS20) | US3 | 9.97E-15 | 1.79E-29 | 1.13E-31 | NS | 1.14E-06 | 8.09E-08 |
| 60 | US5 | 1015 | 1619 | Unknown(US10) | IS12 | 3.62E-08 | 8.20E-18 | 6.55E-22 | 8.96E-07 | 7.25E-04 | 9.08E-12 |
| 61 | US6 | 289 | 1709 | IS2 | US8 | 1.18E-33 | 4.04E-69 | 9.16E-43 | 2.55E-22 | 2.84E-13 | 6.64E-18 |
| 62 | US6 | 1167 | 1618 | Unknown(IS12) | US15 | 5.64E-07 | NS | 1.22E-20 | 3.93E-09 | 2.73E-06 | 3.14E-02 |
| 63 | US6 | 40 | 1520 | BuS3 | Unknown(BuS4) | 6.02E-09 | 6.43E-11 | 9.33E-11 | 2.51E-05 | 1.49E-05 | 8.59E-12 |
| 64 | US6 | 2877 | 1641 | Unknown(KS20) | US3 | 9.97E-15 | 1.79E-29 | 1.13E-31 | NS | 1.14E-06 | 8.09E-08 |
| 65 | US7 | 2899 | 1779 | IS2 | US8 | 1.18E-33 | 4.04E-69 | 9.16E-43 | 2.55E-22 | 2.84E-13 | 6.64EE-18 |
| 66 | US7 | 1167 | 1618 | Unknown(IS12) | US15 | 5.64E-07 | NS | 1.22E-20 | 3.93E-09 | 2.73E-06 | 3.14E-02 |
| 67 | US7 | 2877 | 1532 | Unknown(KS20) | US3 | 9.97E-15 | 1.79E-29 | 1.13E-31 | NS | 1.14E-06 | 8.09E-08 |
| 68 | US8 | 1167 | 1618 | Unknown(IS12) | US15 | 5.64E-07 | NS | 1.22E-20 | 3.93E-09 | 2.73E-06 | 3.14E-02 |
| 69 | US8 | 2850 | 1780 | Unknown(US7) | KS3 | 4.73E-36 | 1.83E-75 | 1.93E-41 | 1.87E-05 | 1.92E-15 | 8.06E-23 |
| 70 | US8 | 999 | 2833 | Unknown(US10) | IS12 | 3.62E-08 | 8.20E-18 | 6.55E-22 | 8.96E-07 | 7.25E-04 | 9.08E-12 |
| 71 | US9 | 2921 | 153 | JS1 | US7 | 3.10E-11 | 4.68E-22 | 1.21E-26 | 5.21E-09 | NS | 2.58E-05 |
| 72 | US9 | 2927 | 1790 | Unknown(IS12) | IS7 | 9.95E-109 | 1.21E-106 | 1.67E-109 | 3.65E-27 | 3.40E-27 | 1.85E-30 |
| 73 | US9 | 703 | 1904 | Unknown(US10) | IS12 | 3.62E-08 | 8.20E-18 | 6.55E-22 | 8.96E-07 | 7.25E-04 | 9.08E-12 |
| 74 | US10 | 2765 | 1774 | Unknown(KS20) | US15 | 1.91E-06 | 1.15E-06 | 7.76E-12 | 1.29E-09 | 1.07E-04 | 2.60E-07 |
| 75 | US10 | 160 | 1876 | Unknown(SS3) | SS2 | 2.33E-59 | NS | 3.11E-07 | 8.87E-16 | 1.42E-15 | NS |
| 76 | US10 | 140 | 1616 | BuS3 | BuS2 | 1.74E-06 | NS | 4.49E-14 | 5.71E-12 | 1.36E-04 | 5.96E-20 |
| 77 | US10 | 518 | 1970 | SS1 | SS2 | 2.54E-60 | 3.00E-57 | 3.78E-44 | 5.93E-16 | 1.17E-20 | 2.87E-11 |
| 78 | US11 | 90 | 1618 | IS2 | Unknown(US5) | 4.83E-14 | 8.68E-46 | 2.14E-22 | 1.02E-11 | 2.89E-07 | 2.88E-23 |
| 79 | US11 | 969 | 1523 | Unknown(BS3) | CS2 | 9.40E-05 | 3.13E-08 | 1.14E-06 | 4.83E-05 | 5.27E-06 | 1.13E-06 |
| 80 | US12 | 40 | 1620 | US15 | Unknown(BuS3) | 8.55E-27 | 1.02E-52 | 4.57E-41 | 1.81E-16 | 1.27E-11 | 2.67E-15 |
| 81 | US12 | 2911 | 1618 | US15 | Unknown(US8) | 4.11E-14 | 2.84E-25 | 1.49E-13 | 1.20E-10 | 4.35E-07 | 2.61E-06 |
| 82 | US12 | 25 | 1312 | US15 | Unknown(CS2) | 4.41E-02 | 8.08E-13 | 9.10E-09 | NS | NS | 2.57E-05 |
| 83 | US12 | 140 | 1799 | Unknown(BuS3) | US10 | 4.15E-21 | 4.62E-36 | 3.86E-42 | 8.46E-16 | 1.80E-10 | 2.11E-25 |
| 84 | US12 | 2965 | 1754 | Unknown(KS2) | KS18 | 5.72E-34 | 4.34E-63 | 3.03E-35 | 5.03E-17 | 1.53E-14 | 6.12E-20 |
| 85 | US12 | 1015 | 1466 | US7 | Unknown(IS12) | 1.61E-08 | NS | NS | 1.38E-07 | 3.99E-06 | NS |
| 86 | US13 | 2938 | 1530 | US1 | Unknown(US12) | 3.09E-20 | 1.36E-35 | 5.68E-23 | 1.80E-17 | 2.02E-10 | 5.76E-14 |
| 87 | US13 | 40 | 1620 | US15 | Unknown(US12) | 1.07E-21 | 6.99E-49 | 2.65E-36 | 5.94E-16 | 9.61E-11 | 3.84E-15 |
| 88 | US13 | 2942 | 1759 | KS12 | Unknown(CS4) | 9.42E-21 | 9.24E-42 | 4.97E-31 | 2.34E-12 | 9.11E-09 | 1.63E-12 |
| 89 | US14 | 119 | 1749 | US7 | CS6 | 1.18E-15 | 8.98E-06 | 4.21E-34 | 6.45E-16 | 4.42E-07 | 9.00E-26 |
| 90 | US14 | 2954 | 1560 | Unknown(BuS4) | BuS3 | 6.02E-09 | 6.43E-11 | 9.33E-11 | 2.51E-05 | 1.49E-05 | 9.59E-12 |
| 91 | US14 | 2965 | 1754 | Unknown(KS2) | KS18 | 5.72E-34 | 4.34E-63 | 3.03E-35 | 5.03E-17 | 1.53E-14 | 6.12E-20 |
| 92 | US15 | 117 | 1620 | BuS3 | Unknown(US10) | 5.01E-15 | 2.39E-31 | 1.44E-36 | 1.21E-13 | 4.02E-08 | 2.16E-23 |
| 93 | US15 | 2966 | 1757 | KS18 | Unknown(KS2) | 5.72E-34 | 4.34E-63 | 3.03E-35 | 5.03E-17 | 1.53E-14 | 6.12E-20 |
| 94 | US15 | 1016 | 1631 | US7 | Unknown(IS12) | 1.61E-08 | NS | NS | 1.38E-07 | 3.99E-06 | NS |
| 95 | BuS2 | 2902 | 1670 | BuS4 | US7 | 5.62E-13 | 8.61E-30 | 3.05E-13 | 5.74E-13 | 3.04E-09 | 2.50E-13 |
| 96 | BuS3 | 2943 | 1652 | Unknown(US12) | US1 | 3.09E-20 | 1.36E-36 | 5.68E-23 | 1.80E-17 | 2.02E-10 | 5.76E-14 |
| 97 | BuS3 | 2921 | 1560 | BuS4 | US7 | 1.72E-10 | 1.20E-16 | 9.36E-21 | 4.84E-05 | 6.49E-10 | 1.61E-09 |
| 98 | BuS3 | 1294 | 1708 | CS2 | Unknown(BS3) | 9.40E-05 | 3.13E-08 | 1.14E-06 | 4.83E-05 | 5.27E-06 | 1.13E-06 |
| 99 | BuS4 | 2 | 1627 | Unknown(US12) | US1 | 1.33E-17 | 2.69E-34 | 2.97E-20 | 1.31E-14 | 2.09E-08 | 6.73E-12 |
| 100 | BuS4 | 2950 | 1532 | Unknown(US8) | US3 | 1.26E-07 | 4.13E-14 | NS | 8.12E-11 | NS | NS |
| 101 | BuS4 | 1305 | 1584 | Unknown(BS3) | CS2 | 9.40E-05 | 3.13E-08 | 1.14E-06 | 4.83E-05 | 5.27E-06 | 1.13E-06 |
| 102 | IS2 | 2941 | 1806 | KS12 | Unknown(CS2) | 4.40E-32 | 4.99E-57 | 5.08E-45 | 2.31E-03 | 6.66E-14 | 6.06E-28 |
| 103 | IS3 | 3 | 1237 | IS14 | US4 | 3.50E-62 | 9.05E-60 | 4.90E-63 | 1.42E-26 | 4.11E-15 | 8.04E-17 |
| 104 | IS4 | 192 | 1664 | SS3 | US2 | NS | 2.20E-09 | NS | 8.58E-08 | 2.63E-03 | 3.67E-19 |
| 105 | IS4 | 2894 | 1790 | IS12 | BuS3 | 2.05E-33 | 1.15E-59 | 7.30E-41 | 2.72E-10 | 2.16E-11 | 6.23E-19 |
| 106 | IS4 | 2922 | 1969 | IS8 | US9 | 8.29E-74 | 2.30E-74 | 3.64E-71 | 1.79E-17 | 9.35E-05 | 6.85E-20 |
| 107 | IS4 | 1234 | 1646 | US12 | Unknown(US2) | 6.67E-15 | 6.85E-25 | 3.15E-23 | 1.51E-11 | 3.00E-05 | 1.68E-18 |
| 108 | IS4 | 2927 | 1776 | US10 | BuS3 | 1.50E-24 | 9.50E-42 | 2.59E-45 | 9.08E-20 | 4.78E-13 | 1.03E-26 |
| 109 | IS4 | 20 | 1847 | US9 | IS10 | 1.52E-77 | 2.49E-75 | 3.78E-26 | 5.94E-23 | 1.99E-24 | 4.91E-23 |
| 110 | IS5 | 192 | 1664 | SS3 | US2 | NS | 2.20E-09 | NS | 8.58E-08 | 2.63E-03 | 3.67E-19 |
| 111 | IS5 | 2922 | 1969 | IS8 | US9 | 8.29E-74 | 2.30E-74 | 3.64E-71 | 1.79E-17 | 9.35E-05 | 6.85E-20 |
| 112 | IS5 | 2894 | 1804 | IS12 | BuS3 | 2.05E-33 | 1.15E-59 | 7.30E-41 | 2.72E-10 | 2.16E-11 | 6.23E-19 |
| 113 | IS5 | 1234 | 1646 | US12 | Unknown(US2) | 6.67E-15 | 6.85E-25 | 3.15E-23 | 1.51E-11 | 3.00E-05 | 1.68E-18 |
| 114 | IS5 | 2927 | 1776 | US10 | BuS3 | 1.50E-24 | 9.50E-42 | 2.59E-45 | 9.08E-20 | 4.78E-13 | 1.03E-26 |
| 115 | IS5 | 20 | 1847 | US9 | IS10 | 1.52E-77 | 2.49E-75 | 3.78E-26 | 5.94E-23 | 1.99E-24 | 4.91E-23 |
| 116 | IS5 | 2927 | 1847 | IS12 | US9 | 6.91E-97 | 7.81E-94 | 5.70E-96 | 2.91E-28 | 5.25E-26 | 3.15E-24 |
| 117 | IS6 | 192 | 1664 | SS3 | US2 | NS | 2.20E-09 | NS | 8.58E-08 | 2.63E-03 | 3.67E-19 |
| 118 | IS6 | 2923 | 1967 | IS8 | US9 | 8.29E-74 | 2.30E-74 | 3.64E-71 | 1.79E-17 | 9.35E-05 | 6.85E-20 |
| 119 | IS6 | 2894 | 1804 | IS12 | BuS3 | 2.05E-33 | 1.15E-59 | 7.30E-41 | 2.72E-10 | 2.16E-11 | 6.23E-19 |
| 120 | IS6 | 2927 | 1776 | US10 | BuS3 | 1.50E-24 | 9.50E-42 | 2.59E-45 | 9.08E-20 | 4.78E-13 | 1.03E-26 |
| 121 | IS6 | 20 | 1847 | US9 | IS10 | 1.52E-77 | 2.49E-75 | 3.78E-26 | 5.94E-23 | 1.99E-24 | 4.91E-23 |
| 122 | IS6 | 2927 | 1847 | IS12 | US9 | 6.91E-97 | 7.81E-94 | 5.70E-96 | 2.91E-28 | 5.25E-26 | 3.15E-24 |
| 123 | IS7 | 192 | 1664 | SS3 | US2 | NS | 2.20E-09 | NS | 8.58E-08 | 2.63E-03 | 3.67E-19 |
| 124 | IS7 | 2923 | 1967 | IS8 | US9 | 8.29E-74 | 2.30E-74 | 3.64E-71 | 1.79E-17 | 9.35E-05 | 6.85E-20 |
| 125 | IS7 | 2894 | 1636 | BuS3 | Unknown(IS12) | 2.05E-33 | 1.15E-59 | 7.30E-41 | 2.72E-10 | 2.16E-11 | 6.23E-19 |
| 126 | IS7 | 20 | 1847 | US9 | US10 | 1.52E-77 | 2.49E-75 | 3.78E-26 | 5.94E-23 | 1.99E-24 | 4.91E-23 |
| 127 | IS7 | 2927 | 2847 | IS12 | US9 | 6.91E-97 | 7.81E-94 | 5.70E-96 | 2.91E-28 | 5.25E-26 | 3.15E-24 |
| 128 | IS8 | 538 | 1761 | KS20 | Unknown(KS2) | 1.82E-06 | 4.26E-07 | 1.17E-21 | 1.12E-05 | 1.45E-02 | 5.29E-11 |
| 129 | IS8 | 2907 | 1664 | IS12 | Unknown(US14) | 1.48E-14 | 1.34E-43 | 1.58E-12 | 5.69E-12 | 3.75E-10 | 7.73E-18 |
| 130 | IS8 | 220 | 1585 | IS2 | Unknown(US11) | 3.21E-14 | 5.66E-22 | 8.54E-31 | 6.38E-13 | 8.09E-07 | 1.49E-10 |
| 131 | IS8 | 1159 | 1846 | KS18 | US10 | NS | 182E-10 | 1.51E-11 | NS | NS | 3.83E-12 |
| 132 | IS9 | 2923 | 1970 | IS8 | US9 | 8.29E-74 | 2.30E-74 | 3.64E-71 | 1.79E-17 | 9.35E-05 | 6.85E-20 |
| 133 | IS9 | 2894 | 1255 | BuS3 | Unknown(IS12) | 2.05E-33 | 1.15E-59 | 7.30E-41 | 2.72E-10 | 2.16E-11 | 6.23E-19 |
| 134 | IS9 | 1312 | 1810 | Unknown(IS12) | IS5 | 5.64E-48 | 7.59E-49 | 1.38E-45 | 4.22E-11 | 6.91E-11 | 1.03E-10 |
| 135 | IS9 | 20 | 1833 | IS10 | US9 | 1.52E-77 | 2.49E-75 | 3.78E-26 | 5.94E-23 | 1.99E-24 | 4.91E-23 |
| 136 | IS9 | 132 | 1610 | IS2 | Unknown(US11) | 3.21E-14 | 5.66E-22 | 8.54E-31 | 6.38E-13 | 8.09E-07 | 1.49E-10 |
| 137 | IS10 | 350 | 1664 | IS12 | Unknown(US14) | 1.48E-14 | 1.34E-43 | 1.58E-12 | 5.69E-12 | 3.75E-10 | 7.73E-18 |
| 138 | IS10 | 85 | 1796 | IS9 | Unknown(BuS3) | 6.81E-43 | 5.13E-83 | 2.45E-61 | 3.71E-25 | 8.17E-18 | 1.14E-25 |
| 139 | IS10 | 2929 | 1754 | Unknown(KS2) | KS18 | 5.72E-34 | 4.34E-63 | 3.03E-35 | 5.03E-17 | 1.53E-14 | 6.12E-20 |
| 140 | IS11 | 192 | 1798 | SS3 | US2 | NS | 2.20E-09 | NS | 8.58E-08 | 2.63E-03 | 3.67E-19 |
| 141 | IS11 | 2923 | 1967 | IS8 | US9 | 8.29E-74 | 2.30E-74 | 3.64E-71 | 1.79E-17 | 9.35E-05 | 6.85E-20 |
| 142 | IS11 | 2894 | 1255 | IS12 | Unknown(BuS3) | 2.05E-33 | 1.15E-59 | 7.30E-41 | 2.72E-10 | 2.16E-11 | 6.23E-19 |
| 143 | IS11 | 606 | 1536 | US12 | Unknown(US2) | 6.67E-15 | 6.85E-25 | 3.15E-23 | 1.51E-11 | 3.00E-05 | 1.68E-18 |
| 144 | IS11 | 20 | 1795 | IS10 | US9 | 1.52E-77 | 2.49E-75 | 3.78E-26 | 5.94E-23 | 1.99E-24 | 4.91E-23 |
| 145 | IS12 | 2926 | 1788 | IS7 | IS3 | 1.41E-23 | 6.10E-79 | 5.09E-141 | 1.27E-20 | 6.14E-22 | 7.39E-25 |
| 146 | IS12 | 1159 | 1938 | KS18 | US10 | NS | 1.82E-10 | 1.51E-11 | NS | NS | 3.82E-12 |
| 147 | IS13 | 2919 | 1177 | IS1 | Unknown(BS3) | 8.86E-05 | 1.67E-15 | 3.39E-17 | 6.00E-06 | 1.86E-02 | 6.33E-10 |
| 148 | IS13 | 2958 | 1867 | IS2 | CS1 | 3.97E-21 | 1.04E-35 | 1.46E-34 | NS | NS | 2.29E-21 |
| 149 | IS14 | 192 | 1664 | SS3 | US2 | NS | 2.20E-09 | NS | 8.58E-08 | 2.63E-03 | 3.67E-19 |
| 150 | IS14 | 2923 | 1967 | IS8 | US9 | 8.29E-74 | 2.30E-74 | 3.64E-71 | 1.79E-17 | 9.35E-05 | 6.85E-20 |
| 151 | IS14 | 2894 | 1636 | BuS3 | Unknown(IS12) | 2.05E-33 | 1.15E-59 | 7.30E-41 | 2.72E-10 | 2.16E-11 | 6.23E-19 |
| 152 | IS14 | 606 | 1645 | US12 | Unknown(US2) | 6.67E-15 | 6.85E-25 | 3.15E-23 | 1.51E-11 | 3.00E-05 | 1.68E-18 |
| 153 | IS14 | 2927 | 1776 | US10 | BuS3 | 1.50E-24 | 9.50E-42 | 2.59E-45 | 9.08E-20 | 4.78E-13 | 1.03E-26 |
| 154 | IS14 | 20 | 1847 | US9 | IS10 | 1.52E-77 | 2.49E-75 | 3.78E-26 | 5.94E-23 | 1.99E-24 | 4.91E-23 |
| 155 | IS14 | 2927 | 1847 | IS12 | US9 | 6.91E-97 | 7.81E-94 | 5.70E-96 | 2.91E-28 | 5.25E-26 | 3.15E-24 |
| 156 | IS15 | 2942 | 1711 | KS12 | Unknown(CS4) | 9.43E-21 | 9.24E-42 | 4.99E-31 | 2.34E-12 | 9.11E-09 | 1.63E-12 |
| 157 | IS16 | 2906 | 750 | IS15 | Unknown(IS11) | 5.99E-26 | 1.15E-21 | NS | 5.39E-06 | 1.98E-07 | NS |
| 158 | SS1 | 2913 | 1583 | Unknown(KS2) | KS20 | 1.82E-06 | 4.26E-07 | 1.17E-21 | 1.12E-05 | 1.45E-02 | 5.29E-11 |
| 159 | SS1 | 488 | 1761 | IS8 | Unknown(US2) | 1.39E-27 | 4.41E-54 | 2.51E--37 | 3.83E-19 | 4.37E-11 | 3.61E-18 |
| 160 | SS1 | 966 | 1784 | SS2 | Unknown(US10) | 1.89E-59 | 6.55E-53 | 4.59E-40 | 1.09E-15 | 2.63E-15 | 2.74E-18 |
| 161 | SS2 | 2922 | 1970 | Unknown(SS1) | US10 | 2.54E-60 | 3.00E-57 | 4.48E-26 | 7.14E-29 | 5.29E-18 | 4.89E-11 |
| 162 | SS2 | 205 | 910 | IS12 | Unknown(US14) | 1.48E-14 | 1.34E-43 | 1.58E-28 | 5.69E-12 | 3.74E-10 | 7.73E-18 |
| 163 | SS2 | 122 | 1800 | SS4 | Unknown(BuS3) | 3.33E-38 | 3.93E-72 | 1.00E-47 | 2.58E-03 | 9.71E-17 | 4.69E-21 |
| 164 | SS2 | 282 | 1625 | US11 | Unknown(IS2) | 3.21E-14 | 5.66E-22 | 8.54E-31 | 6.38E-13 | 8.09E-07 | 1.49E-10 |
| 165 | SS2 | 1287 | 1876 | SS1 | US10 | 1.57E-57 | 7.85E-52 | 5.42E-52 | 6.40E-13 | 2.98E-13 | 2.61E-17 |
| 166 | SS3 | 2770 | 1784 | Unknown(KS20) | US15 | 1.91E-06 | 1.15E-06 | 7.76E-12 | 1.29E-09 | 1.07E-04 | 2.60E-07 |
| 167 | SS3 | 1234 | 1761 | IS8 | Unknown(US2) | 1.39E-27 | 4.41E-54 | 2.51E--37 | 3.83E-19 | 4.37E-11 | 3.61E-18 |
| 168 | SS3 | 569 | 1885 | SS2 | Unknown(US10) | 1.89E-59 | 6.55E-53 | 4.59E-40 | 1.09E-15 | 2.63E-15 | 2.74E-18 |
| 169 | SS3 | 78 | 1622 | Unknown(US11) | IS2 | 3.21E-14 | 5.66E-22 | 8.54E-31 | 6.38E-13 | 8.09E-07 | 1.49E-10 |
| 170 | SS4 | 2770 | 1784 | Unknown(KS20) | US15 | 1.91E-06 | 1.15E-06 | 7.76E-12 | 1.29E-09 | 1.07E-04 | 2.60E-07 |
| 171 | SS4 | 1234 | 1761 | IS8 | Unknown(US2) | 1.39E-27 | 4.41E-54 | 2.51E--37 | 3.83E-19 | 4.37E-11 | 3.61E-18 |
| 172 | SS4 | 78 | 1622 | Unknown(US11) | IS2 | 3.21E-14 | 5.66E-22 | 8.54E-31 | 6.38E-13 | 8.09E-07 | 1.49E-10 |
| 173 | GS1 | 20 | 907 | US13 | Unknown(US14) | 2.16E-13 | 4.72E-15 | 1.66E-18 | 2.57E-07 | 1.05E-07 | 1.48E-12 |
| 174 | BS1 | 334 | 792 | BuS2 | Unknown(BS2) | 5.63E-20 | 3.42E-19 | NS | 1.69E-04 | 1.52E-04 | NS |

NS: 175not significant
