# Supplementary material for: Identification of three new isolates of Tomato spotted wilt virus from different hosts in China: molecular diversity, phylogenetic and recombination analyses
Source: Virol J. 2016 Jan 14;13:8. doi: 10.1186/s12985-015-0457-3 (PMC4712509; doi:10.1186/s12985-015-0457-3)
Supplement: Additional file 5: Table S5. — Primers used in this study. Primers were designed based on previously reported TSWV sequences. W: A/T, M: A/C, R: G/A, Y: C/T. (DOCX 15 kb) [file 12985_2015_457_MOESM5_ESM.docx]

Table S5. Primers used in this study

| Primer name | Sequences (5’⭢3’) | polarity | purpose |
| --- | --- | --- | --- |
| TS-S-5’-F | AGAGCAATTGTGTCATAATTTTATTCWTAATCAAACCTCAC | + | Cloning of TSWV-S |
| TS-S-3’-R | AGAGCAATTGTGTCAATTTTATTCAAACCTTAAMAC | - |  |
| TS-S-602-F | GTCGAGATGTGCTACAATCAAG | + | Sequencing of TSWV-S |
| TS-S-2321-R | GAAGTATGACACCAGGGAAGCC | - |  |
| TS-M-5’-F | AGAGCAATCAGTGCRTCAGAAATATACCTATTATACAYTTTGC | + | Cloning of TSWV-M fragment (1-3401) |
| TS-M-3401-R | GTTCCATCTAATAGTGAACACTAAGC | - |  |
| TS-M-2215-F | CAGGATCATTCAAGTTTGCAATATTTC | + | Cloning of TSWV-M fragment(2215-4772) |
| TS-M-3’-R | AGAGCAATCAGTGCAAACAAAAACCTTAATCCAGAC | - |  |
| TS-M-700-F | GATGTGCAGCCAAGAATACAAG | + | Sequencing of TSWV-M |
| TS-M-2281-R | GAACTGGTAGCTCATGGACC | - |  |
| TS-M-3299-F | CATAGACATGGGCATTTGAGAC | + |  |
| TS-L-5’-F | AGAGCAATCAGGTAACAACGATTTTAAGCAAACATGAACATC | + | Cloning of TSWV-L fragment (1-2722) |
| TS-L-2722-R | GCTTTGTCACAGTTCCTATC | - |  |
| TS-L-2512-F | CTCAATCAGGTGAGGCTGCTAAG | + | Cloning of TSWV-L fragment(2512-5927) |
| TS-L-5927-R | GAGCCTTCAACAACAGGTATCC | - |  |
| TS-L-5906-F | GGATACCTGTTGTTGAAGGCTC | + | Cloning of TSWV-L fragment (5906-8913) |
| TS-L-3’-R | AGAGCAATCAGGTACAACTAAAACATATAATCTCTCC | - |  |
| TS-L-515-F | CTAACGCCACACCTGACAAC | + | Sequencing of TSWV-L |
| TS-L-2103-R | CTTGCCTTCCTCTCTTATATCACC | - |  |
| TS-L-4529-F | CATTGATAGCCAGTGGAGAGGTCG | + |  |
| TS-L-4918-R | CATTCACTTCACCAGGAAGCATTG | - |  |
| TS-L-3103-F | CCTGCTGAGTGGGAGCTAAAG | + |  |
| TS-L-4552-R | CGACCTCTCCACTGGCTATC | - |  |
| TS-L-6364-F | GCTCAGCAAGACATGAACTTAG | + |  |
| TS-L-7021-F | AAGGTTAATGCCACACCAAGAC | + |  |
| TS-L-8224-R | CACTGGAACTTAATCTGCCCA | - |  |

Note:

Primer designed based on the alignment results of previously reported TSWV sequences. W: A/T, M:A/C, R: G/A, Y:C/T
